# Overcoming the entropy of polymer chains by making a plane with terminal groups: a thermoplastic PDMS with a long-range 1D structural order†

**DOI:** 10.1039/d2sc05491d

**Published:** 2023-01-04

**Authors:** Yugen Chen, Fumitaka Ishiwari, Tomoya Fukui, Takashi Kajitani, Haonan Liu, Xiaobin Liang, Ken Nakajima, Masatoshi Tokita, Takanori Fukushima

**Affiliations:** a Laboratory for Chemistry and Life Science, Institute of Innovative Research, Tokyo Institute of Technology 4259 Nagatsuta, Midori-ku Yokohama 226-8503 Japan; b Department of Chemical Science and Engineering, Tokyo Institute of Technology 4259 Nagatsuta, Midori-ku Yokohama 226-8503 Japan ishiwari@chem.eng.osaka-u.ac.jp; c Open Facility Development Office, Open Facility Center, Tokyo Institute of Technology 4259 Nagatsuta, Midori-ku Yokohama 226-8503 Japan; d Department of Chemical Science and Engineering, Tokyo Institute of Technology 2-12-1 Ookayama, Meguro-ku Tokyo 152-8550 Japan; e Living Systems Materialogy (LiSM) Research Group, International Research Frontiers Initiative (IRFI), Tokyo Institute of Technology 4259 Nagatsuta, Midori-ku Yokohama 226-8503 Japan

## Abstract

Due to its unique physical and chemical properties, polydimethylsiloxane (PDMS) is widely used in many applications, in which covalent cross-linking is commonly used to cure the fluidic polymer. The formation of a non-covalent network achieved through the incorporation of terminal groups that exhibit strong intermolecular interactions has also been reported to improve the mechanical properties of PDMS. Through the design of a terminal group capable of two-dimensional (2D) assembly, rather than the generally used multiple hydrogen bonding motifs, we have recently demonstrated an approach for inducing long-range structural ordering of PDMS, resulting in a dramatic change in the polymer from a fluid to a viscous solid. Here we present an even more surprising terminal-group effect: simply replacing a hydrogen with a methoxy group leads to extraordinary enhancement of the mechanical properties, giving rise to a thermoplastic PDMS material without covalent cross-linking. This finding would update the general notion that less polar and smaller terminal groups barely affect polymer properties. Based on a detailed study of the thermal, structural, morphological and rheological properties of the terminal-functionalized PDMS, we revealed that 2D assembly of the terminal groups results in networks of PDMS chains, which are arranged as domains with long-range one-dimensional (1D) periodic order, thereby increasing the storage modulus of the PDMS to exceed its loss modulus. Upon heating, the 1D periodic order is lost at around 120 °C, while the 2D assembly is maintained up to ∼160 °C. The 2D and 1D structures are recovered in sequence upon cooling. Due to the thermally reversible, stepwise structural disruption/formation as well as the lack of covalent cross-linking, the terminal-functionalized PDMS shows thermoplastic behavior and self-healing properties. The terminal group presented herein, which can form a ‘plane’, might also drive other polymers to assemble into a periodically ordered network structure, thereby allowing for significant modulation of their mechanical properties.

## Abstract 2

Subtle modification, adding methoxy groups to the triptycene termini of a telechelic polydimethylsiloxane (PDMS), dramatically improves mechanical properties, forming a thermoplastic PDMS without the need for covalent cross-linking.

## Graphical abstract

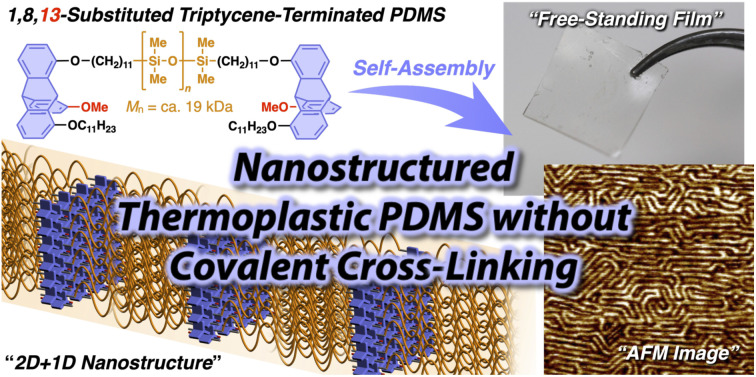

## Introduction

Incorporation of functional groups capable of exhibiting strong intermolecular interactions into the termini of polymers can result in improvement in the mechanical and thermal properties of polymers.^1–9^ Since the pioneering work by Meijer and co-workers, which demonstrated a striking effect of 2-ureido-4-pyrimidone as polymer termini that can form multiple hydrogen bonds^10,11^ to improve the mechanical properties of polymers, various terminal groups that exhibit hydrogen bonding, metal coordination, ion pairing, or host–guest interaction have been developed.^1–9^ Originally, the approach of using such terminal functional groups was aimed at linking polymer chains linearly through noncovalent interactions, to increase the apparent molecular weights. Multiple noncovalent interactions between polymer termini or side chains have recently been used to achieve the cross-linking of polymer chains to create a network structure, thereby increasing the mechanical properties of polymers more than in the case of conventional linearly linked polymers.^1–9,12,13^

Another interesting prospect in the design of terminal groups is the possibility of inducing the controlled assembly of polymers into a higher-order hierarchical structure.^1–9^ Such ordered polymer assemblies could lead to applications in nanopatterning and directional materials transport.^14–17^ Nonetheless, as the weight fraction of terminal groups relative to the polymer main chain is considerably low, the formation of a higher-order structure of polymers by terminal functionalization is generally difficult to achieve, and successful examples have been limited to relatively low molecular weight polymers (*M*_n_ < *ca*. 8 kDa) with a narrow molecular weight distribution (*Đ*).^18–23^ In some cases, a stepwise synthesis of discrete oligomers with *Đ* = 1 is required to create a higher-order polymer assembly.^24–28^

**Fig. 1:**
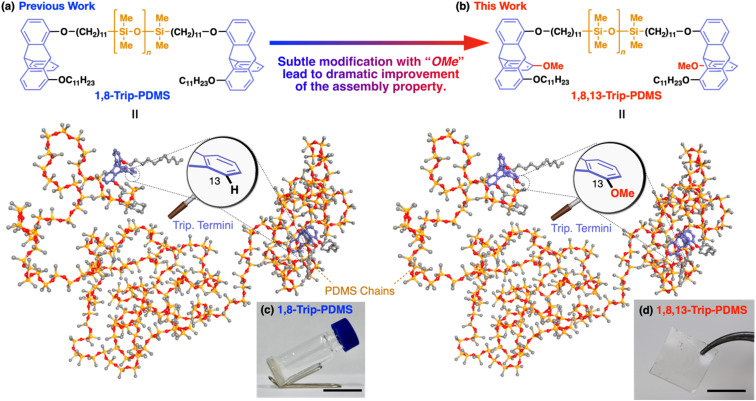
Chemical structures and 3D models (240 mer, *M*_n_ = *ca.* 19 kDa) of (a) 1,8-Trip-PDMS and (b) 1,8,13-Trip-PDMS. Photographs of samples at 25 °C of (c) 1,8-Trip-PDMS (a viscous solid) and (d) 1,8,13-Trip-PDMS (a free-standing film). Scale bars = 1 cm.

We previously reported that polydimethylsiloxanes (PDMSs) with a molecular weight (*M*_n_) of 18–24 kDa, bearing a triptycene unit (1,8-Trip-PDMS, Fig. 1a) at both termini, show remarkable improvements in mechanical and thermal properties, compared with the corresponding hydride-terminated PDMSs.^29^ The design of the triptycene-terminated PDMSs relied on the finding that 1,8,13-substituted and 1,8-substituted triptycenes can self-assemble into a well-defined “2D + 1D” structure with exceptionally long-range order,^30–33^ where 2D arrays, formed by nested hexagonal packing of the triptycene, stack into a 1D layer structure. The structuring ability of 1,8,13- and 1,8-substituted triptycenes was also found to work well for polymeric materials.^34,35^ Thus, 1,8-Trip-PDMS self-assembles to form a highly-ordered “2D + 1D” structure with a layer spacing of 18–20 nm despite its large molecular weight distribution (*Đ* ≈ 2).^29^ Consequently, although the precursor hydride-terminated PDMS is a fluid, the PDMSs with the triptycene termini (1,8-Trip-PDMS, Fig. 1a) turns into a viscous solid (Fig. 1c) with a dramatic increase in complex viscosity by four orders of magnitude.

The above finding encouraged us to further investigate the terminal-group effect on the mechanical and thermal properties of PDMS using a 1,8,13-substituted triptycene unit with a methoxy group at the 13-position (1,8,13-Trip-PDMS, Fig. 1b). The rationale for changing from 1,8-substituted to 1,8,13-substituted triptycene is based on the fact that 1,8-bis(dodecyloxy)-13-methoxytriptycene exhibits a much higher melting point (231 °C)^30^ than 1,8-bis(dodecyloxy)triptycene (134 °C, Fig. S1†).^33^ This may reflect a difference in structural integrity between the di- and trisubstituted systems. Surprisingly, the presence of a tiny methoxy group on the terminal triptycene, which is indeed very subtle relative to the molecular weight of the entire polymer (*ca.* 0.3 wt%), was found to have a significant impact on the mechanical and thermal properties, resulting in solidification of the inherently liquid PDMS, to allow the formation of a free-standing film without any covalent cross-linking (Fig. 1d). Here we report the terminal group-induced structuring behavior of 1,8,13-Trip-PDMS, as well as its thermal, mechanical and rheological properties. We also describe the self-healing behavior of 1,8,13-Trip-PDMS as a non-covalently crosslinked PDMS material.

## Results and discussion

### Synthesis and characterization of 1,8,13-Trip

**Fig. 2:**
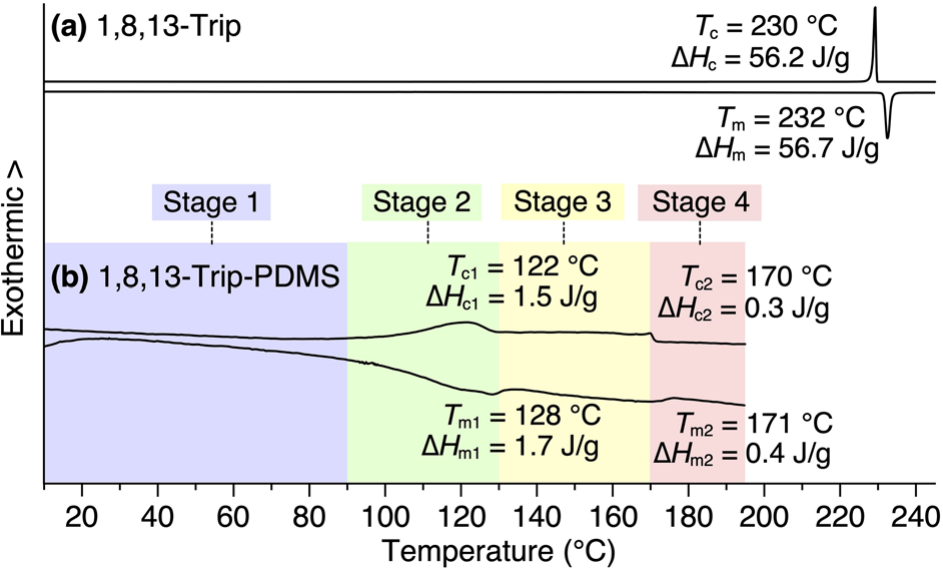
DSC profiles of (a) 1,8,13-Trip and (b) 1,8,13-Trip-PDMS in a second heating/cooling cycle, measured at a scan rate of 10 °C min^−1^ under N_2_ flow (50 mL min^−1^). In (b), the temperature ranges of Stages 1, 2, 3 and 4 for the polymer are pastel-color coded blue, green, yellow and pink, respectively (see also Fig. 5a).

**Fig. 3:**
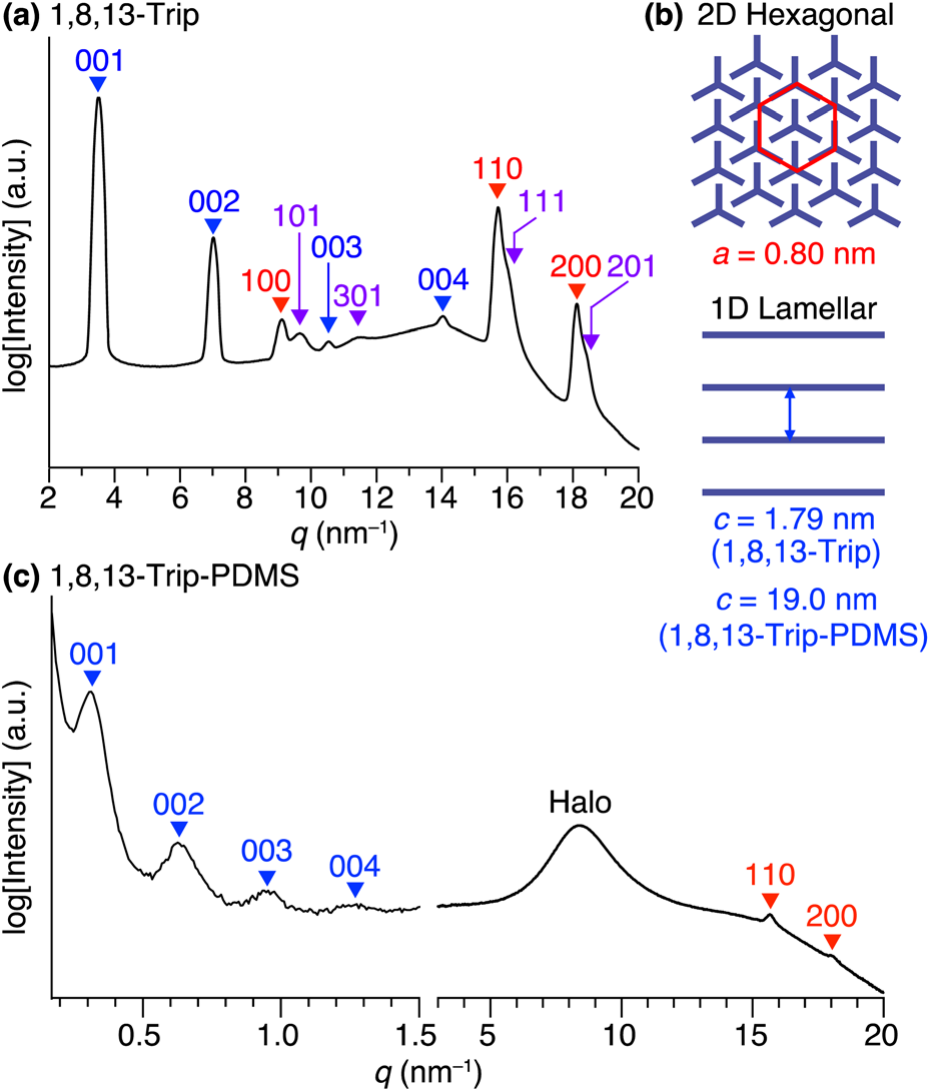
(a) Powder XRD pattern of 1,8,13-Trip at 25 °C measured after cooling from its isotropic liquid in a glass capillary with a diameter of 1.5 mm. (b) Schematic illustrations of a 2D hexagonal array and a 1D lamellar structure formed in the assembly of 1,8,13-Trip. (c) Small- and wide-angle XRD patterns of 1,8,13-Trip-PDMS at 30 °C measured after cooling from its isotropic liquid in a glass capillary with a diameter of 1.5 mm.

Using procedures similar to those reported previously,^29,30^ 1,8,13-Trip (Scheme S1†) to be attached to the termini of PDMS was synthesized by a sequential Williamson etherification of 1,8,13-trihydroxytriptycene 1 with iodomethane,^30^ 11-bromo-1-undecene and 1-bromoundecane. The chemical composition of 1,8,13-Trip was unambiguously characterized by ^1^H NMR and IR spectroscopy and APCI-TOF mass spectrometry (Fig. S2–13†). Differential scanning calorimetry (DSC) showed that the melting (*T*_m_) and crystallization (*T*_c_) temperatures of 1,8,13-Trip (*T*_m_ = 232 °C and *T*_c_ = 230 °C, Fig. 2a) were notably higher than those of 1,8-Trip (*T*_m_ = 134 °C and *T*_c_ = 127 °C).^29^ The powder X-ray diffraction (XRD) pattern of 1,8,13-Trip (Fig. 3a), measured after being heated once to melting temperature and then cooled to 25 °C, is typical of triptycene derivatives that form a “2D + 1D” structure. Thus, the diffractions observed were fully indexed by assuming a hexagonal unit cell with lattice parameters of *a* = 0.80 nm and *c* = 1.79 nm for 1,8,13-Trip (Fig. 3b, Table S1†).

### Synthesis and characterization of 1,8,13-Trip-PDMS

For the synthesis of 1,8,13-Trip-PDMS (Fig. 1b), 1,8,13-Trip was reacted with commercially available hydride-terminated PDMS (H-PDMS, number-averaged molecular weight *M*_n_ = 18 kDa) in toluene in the presence of Karstedt's catalyst (Scheme S1†).^29^ Comparison of the ^1^H NMR and IR spectra of H-PDMS and 1,8,13-Trip-PDMS confirmed that the termini of H-PDMS are completely functionalized with the 1,8,13-Trip group after the hydrosilylation reaction (Fig. S14–S17†). Based on NMR and SEC analysis, the *M*_n_ and *Đ* of 1,8,13-Trip-PDMS were determined to be *M*_n_ = 19.4 kDa and *Đ* = 2.0 (Fig. S16 and S18†). Surprisingly, 1,8,13-Trip-PDMS was obtained as a solid, hard enough to form a free-standing film at 25 °C (Fig. 1d). This is in sharp contrast to previously reported 1,8-Trip-PDMS, which is a viscous solid.^29^

The DSC profile of 1,8,13-Trip-PDMS (Fig. 2b) showed two sets of melting/crystallizing features at lower (*T*_m1_/*T*_c1_) and higher (*T*_m2_/*T*_c2_) temperature regions. Notably, despite a slight difference in the structure of triptycene termini, the *T*_m1_/*T*_c1_ and *T*_m2_/*T*_c2_ temperatures of 1,8,13-Trip-PDMS (around 120 °C and 170 °C, respectively) were much higher than those previously reported for 1,8-Trip-PDMS (around 40 °C and 90 °C, respectively).^29^ The structural properties of 1,8,13-Trip-PDMS are roughly classified into four stages: pastel-color coded blue, green, yellow and pink, respectively. Thermogravimetric analysis showed that the temperature of 1% weight loss was 357 °C, indicating that 1,8,13-Trip-PDMS has high thermal stability (Fig. S19†). Fig. 3c shows the small- and wide-angle XRD patterns of 1,8,13-Trip-PDMS at 30 °C, which are almost identical to those observed for 1,8-Trip-PDMS.^29^ In the wide-angle region, two peaks observed at *q* = 15.7 and 18.5 nm^−1^ are assigned to diffraction from the (110) and (200) planes of a 2D hexagonal array with a lattice parameter (*a*) of 0.8 nm (Fig. 3b), which is formed by nested packing of the triptycene termini. In the small-angle region, 1,8,13-Trip-PDMS exhibited multiple diffraction peaks up to fourth-order from a 1D lamellar structure with layer spacings of 19.0 nm. It is considered that the soft PDMS chains are folded and exist between the layers.

**Fig. 4:**
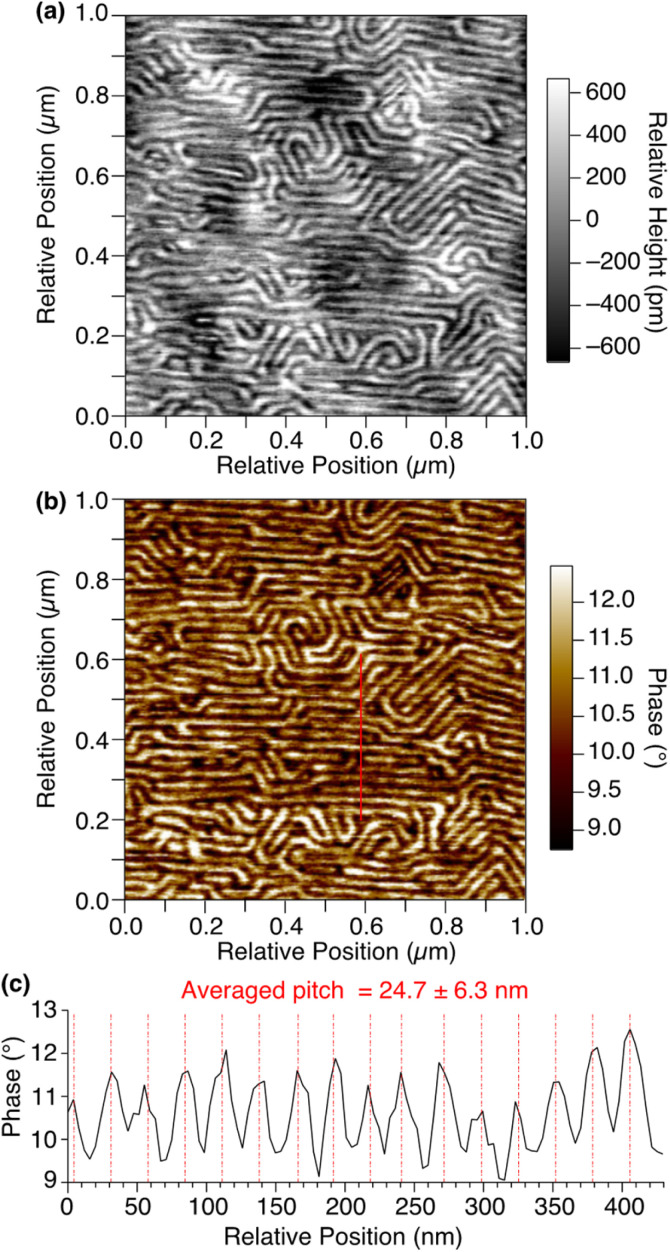
AFM (a) height and (b) phase images of a thin film of 1,8,13-Trip-PDMS on a Si wafer. (c) Phase profile along the red line in (b).

By means of atomic force microscopy (AFM), the ordered assembly structure of 1,8,13-Trip-PDMS was successfully visualized (Fig. 4). To prepare a thin-film sample for AFM observation, a THF solution of 1,8,13-Trip-PDMS (10 mg mL^−1^) was spin-coated (1000 rpm) on a Si wafer at 25 °C, heated to 200 °C under vacuum, and then cooled to 25 °C at a rate of 1 °C min^−1^. The height and phase images of the thin film clearly shows a regular stripe pattern with an average pitch of approximately 25 nm (Fig. 4), which is reminiscent of microphase-separated structures of block copolymers. We presume that the deviation in the pitch from the layer spacing observed by small-angle XRD (*ca*. 19 nm) for a bulk sample might be caused by the influence of the substrate such as a flattening effect.^36–38^ Notably, the uniform and well-ordered structure can be constructed from PDMS with a large molecular-weight distribution (*Đ* = 2.0) only by terminal functionalization with triptycene units, for which strong intermolecular interactions such as multiple hydrogen bonds are not expected.

### Temperature-dependence of the structure-rheological property relationship of 1,8,13-Trip-PDMS

**Fig. 5:**
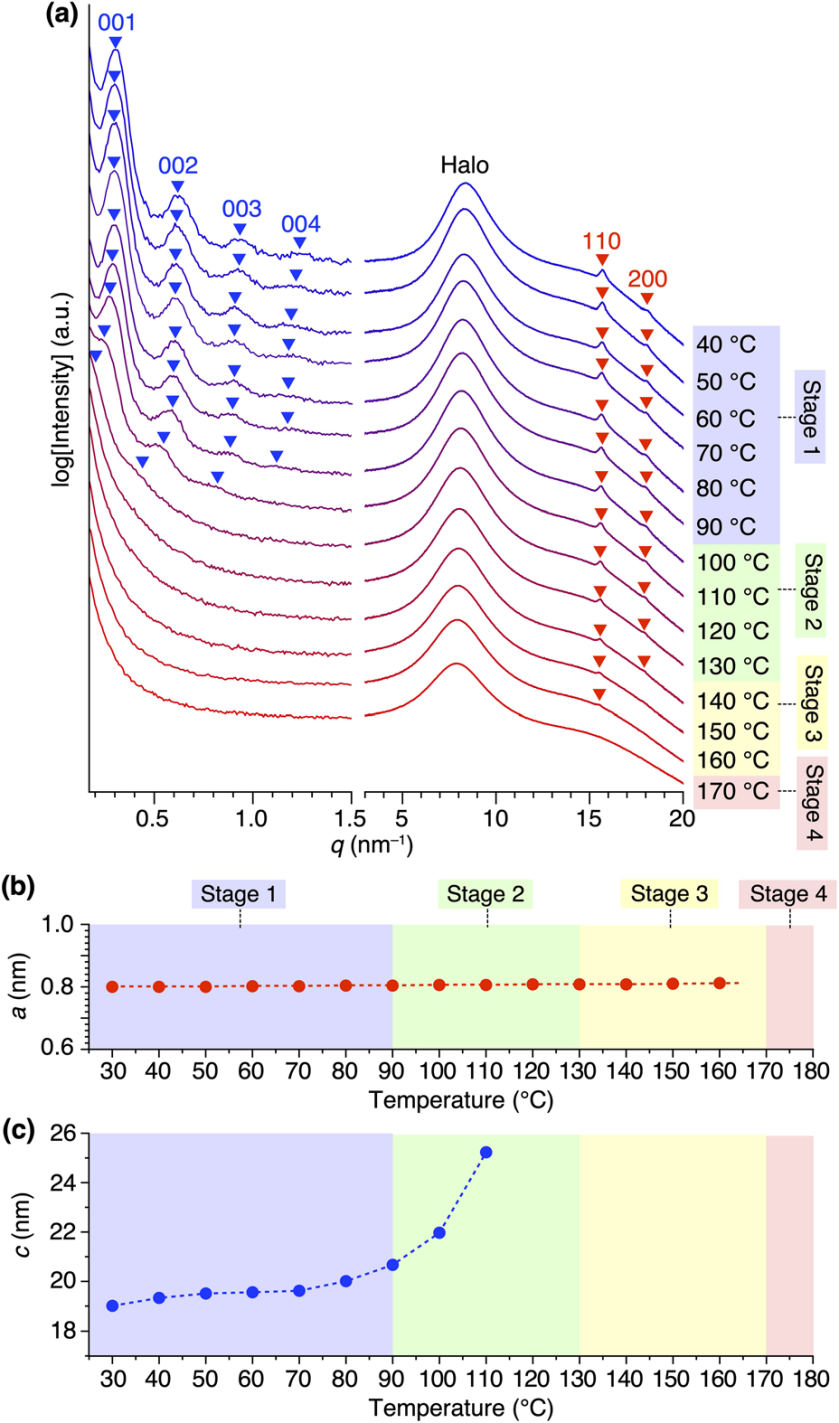
(a) Variable-temperature small- and wide-angle XRD patterns of 1,8,13-Trip-PDMS measured upon heating in a glass capillary with a diameter of 1.5 mm. For magnified profiles, please see Fig. S20.† Right panels represent the temperature ranges of Stages 1–4 in Fig. 2b. Temperature-dependence of (b) 2D hexagonal lattice parameter and (c) 1D layer spacing.

Variable-temperature small- and wide-angle XRD experiments gave insight into the origin of the two melting/crystallizing features of 1,8,13-Trip-PDMS, as well as the structural aspects in the four temperature regions (Stages 1–4) in Fig. 2b. As expected, the powder XRD patterns of 1,8,13-Trip-PDMS were almost unchanged upon heating from 30 to 90 °C (Fig. 3c and 5a). When heated above 90 °C, the 00*n* diffractions due to the 1D lamellar shifted to a smaller *q* region, broadened, and disappeared at 130 °C. In contrast, the 110 and 200 diffractions due to the 2D hexagonal triptycene array were maintained up to 130 °C. Although further heating resulted in a gradual decrease in intensity of these peaks, the 110 diffraction was still detectable even at 160 °C, and it eventually disappeared at 170 °C. The changes in 2D hexagonal lattice parameter *a* and 1D layer spacing *c* with increasing temperature (Fig. 5b and c) represent well the difference in thermal stability between the 2D and 1D structural orders of the 1,8,13-Trip-PDMS assembly. Since the VT-XRD profiles observed upon heating (Fig. 5) and cooling (Fig. S21†) are almost identical, the structuring of 1,8,13-Trip-PDMS is thermally reversible.

**Fig. 6:**
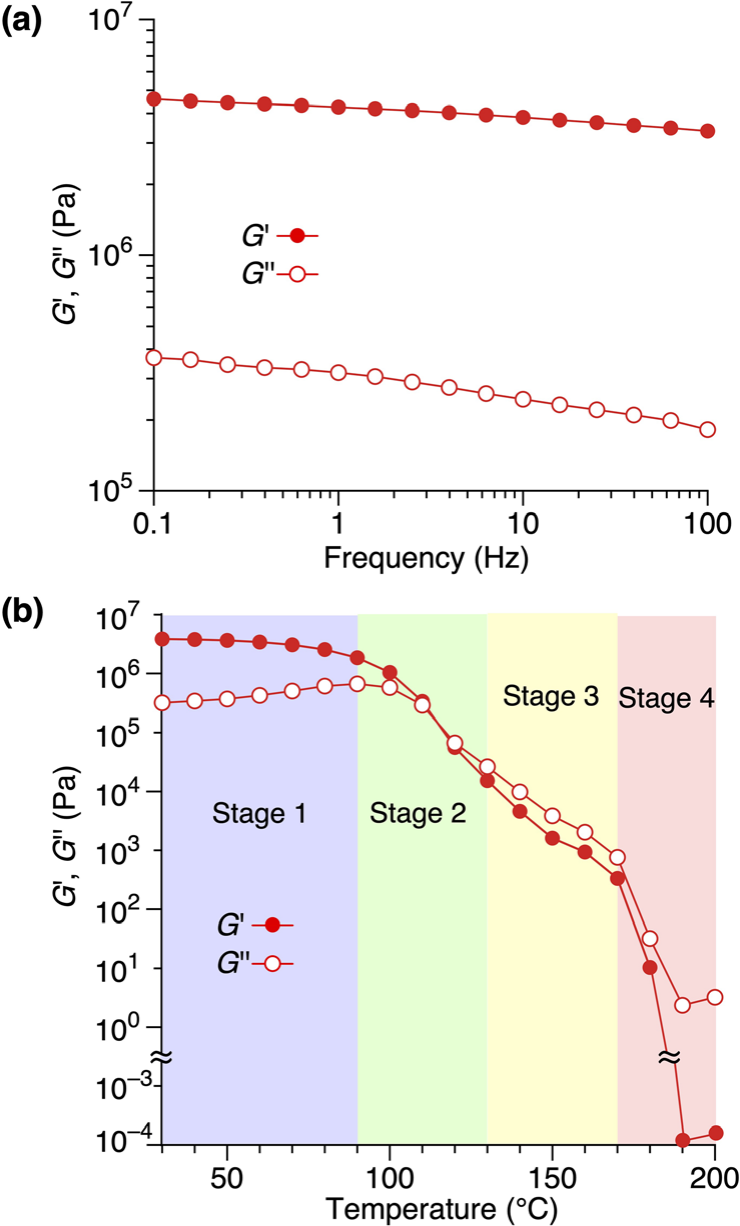
Rheological properties of 1,8,13-Trip-PDMS. (a) Frequency-dependance of *G*′ and *G*′′ at 30 °C. (b) Temperature-dependance of *G*′ and *G*′′ at 1.0 Hz. The temperature ranges of the four Stages are pastel-color coded blue, green, yellow and red, respectively.

Rheological measurements revealed the relationship between the structure and mechanical properties of 1,8,13-Trip-PDMS. The frequency-dependence of storage (*G*′) and loss (*G*′′) moduli of 1,8,13-Trip-PDMS at 30 °C (Fig. 6a)^39^ displayed that the *G*′ values are at a plateau and at a much higher level than the *G*′′ values over the entire range of frequency examined, confirming the solid nature of 1,8,13-Trip-PDMS. Fig. 6b shows the temperature-dependence of *G*′ and *G*′′ of 1,8,13-Trip-PDMS at a frequency of 1.0 Hz.^40^ In a temperature range of 30–90 °C, the *G*′ values are significantly higher than the *G*′′ values, meaning that 1,8,13-Trip-PDMS can maintain its mechanical properties as a shape-persistent solid material. At a temperature range of 100–120 °C, the *G*′ values drop to a level similar to the *G*′′ values, and both decrease rapidly with increasing temperature. Above 130 °C, the *G*′′ values become higher than the *G*′ values, and above 170 °C, both *G*′ and *G*′′ values dramatically decrease to ∼100 Pa as the polymer turns to an isotropic liquid.

**Fig. 7:**
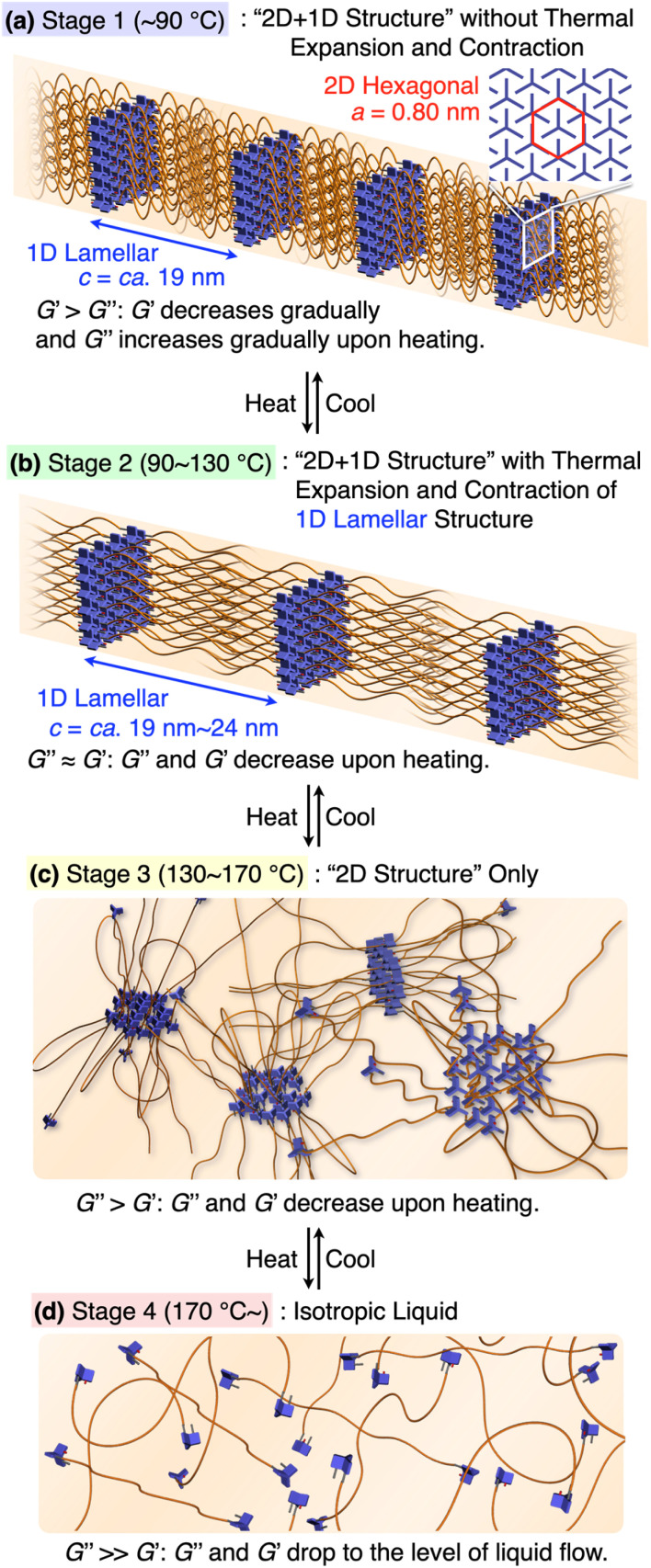
(a–d) Schematic illustration of the assembly structures and viscoelastic properties of 1,8,13-Trip-PDMS associated with the sequential structural change.

Based on the results from the XRD and rheological measurements, we here provide the most plausible scenario that can correlate the structures and rheological properties of 1,8,13-Trip-PDMS in Stages 1–4 (Fig. 7). In the temperature region of Stage 1 (pastel blue), the 2D + 1D structure remains intact. Upon transition to Stage 2 (pastel green), the 1D layer spacing is increased by thermal expansion of the PDMS domain, whereas the structural integrity of the 2D hexagonal array is still maintained, indicating that anisotropic thermal expansion occurs at a nanoscopic scale. Considering that the thermal expansion coefficient of PDMS is approximately 300 ppm K^−1^,^41^ the change in the layer spacing of the 1D lamellar in Stage 2 with increasing temperature appears to be too large (Fig. 5c). Most likely, the 2D triptycene array partially collapses, resulting in the large expansion of the 1D lamellar spacing. In fact, the temperature-dependence of the XRD diffraction intensity from the (110) plane of 1,8,13-Trip-PDMS showed a gradual decrease with increasing temperature (Fig. S20†), indicating that the crystallite coherent length of the 2D triptycene array decreased. When the 2D triptycene array, which serves as a “wall” to accommodate the amorphous PDMS domain, partially collapses upon heating, the motility of the PDMS chain increases further, expanding the interlayer spacing. In Stage 3 (pastel yellow), the 1D lamellar structure completely disappears. Even in this stage, the 2D hexagonal structure of the triptycene units persists. However, above 160 °C, it collapses into an isotropic liquid. While the assembly structure and thermal behavior of 1,8,13-Trip-PDMS are virtually identical to those of previously reported 1,8-Trip-PDMS, the temperature at which each structural change occurs is largely shifted to a much higher region. The observation that a slight chemical modification of only two terminal triptycene units can result in such large structural robustness and thermal stability, along with a change in the material state from a viscous solid (Fig. 1c) to a hard solid (Fig. 1d), was far beyond our expectation.

It is also interesting to note the correlation between the temperature region where *G*′ drops to the level of *G*′′ and eventually reverses, and the structural changes characterized by XRD. We had thought that the elastic properties of 1,8,13-Trip-PDMS were mainly due to the 2D triptycene array. However, considering the fact that such rheological behavior is observed in the temperature range where the 2D array remains but long-range 1D order disappears, the 1D lamellar structure plays a vital role in the elastic properties of the polymer. Importantly, the thermal and rheological properties of 1,8,13-Trip-PDMS were completely reversible in a heating/cooling cycle, providing 1,8,13-Trip-PDMS with a thermoplastic nature.

### Comparison of previously reported thermoplastic PDMSs and 1,8,13-Trip-PDMS

Thermoplastic PDMSs have previously been developed using an ABA-type triblock copolymer system consisting of hard isotactic polystyrene segments (A) with a large volume fraction (>37 wt%) to the PDMS segment (B),^42^ a telechelic polymer or random copolymer system having multiple functional groups capable of strong non-covalent interactions such as hydrogen bonding^12^ or ionic interactions.^43,44^ Very recently, Yao *et al.* reported interesting results,^12^ which demonstrate that PDMS, bearing ureidocytosine (UCy) units with strong multiple-hydrogen bonding and π-stacking ability at both termini, can form an extensive non-covalent network to achieve excellent thermo-mechanical properties. The key design concept is that the enthalpic gains from the aggregation of the UCy units can compensate for the entropic loss from redistribution of the PDMS chains and stabilize the non-covalent networks over a wide temperature range.

Polymers with molecular units that undergo non-covalent 1 : 1 association at both termini can form linear supramolecular polymers.^1–9^ In contrast, terminal functionalization using molecular units that enable non-covalent 1 : *n* (*n* > 2) association would, in principle, give rise to supramolecular polymers with a highly branched polymer chain, resulting in the formation of a non-covalent network structure. As demonstrated by Yao *et al.*,^12^ the formation of such a polymer network would be critical for achieving a dramatic improvement in the mechanical and thermal properties of polymer assemblies. In light of this notion, the 1,8,13-Trip motif is a new class of terminal group, which features the ability to assemble into an infinite and ordered 2D assembly, in which numerous triptycene molecules are engaged, thereby allowing for the formation of polymer chain networks. Furthermore, the polymer chains in the network can align into a higher hierarchical structure with long-range 1D periodic order. Although the triptycene motif does not appear to exhibit strong intermolecular interactions, the formation of the infinite 2D hexagonal array of numerous triptycene molecules results in an enthalpy gain sufficient to compensate for the entropy loss of the PDMS chain even at a high temperature. Therefore, 1,8,13-Trip-PDMS can remain in the solid state over a wide temperature range. The difference in structural and thermal properties between previously reported 1,8-Trip-PDMS and the present polymer reflects the integrity of their assembly structures at the monomer level. This is clearly represented by the large difference in melting point: 232 °C and 134 °C for 1,8,13-Trip and 1,8-Trip, respectively.^29^

**Fig. 8:**
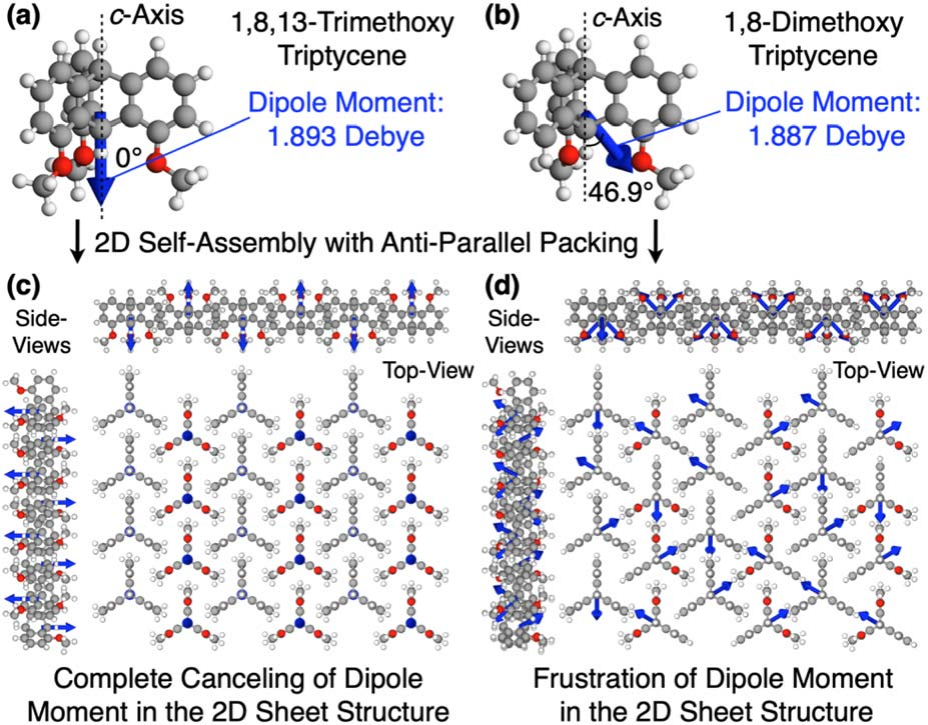
Molecular dipole moments (blue arrows) of (a) 1,8,13-trimethoxy and (b) 1,8-dimethoxy triptycenes obtained by DFT calculations. Models of the assembly structures of (c) 1,8,13-trimethoxy and (d) 1,8-dimethoxy triptycenes, where the dipole moment of each molecule is denoted with a blue arrow.

Why can the replacement of a hydrogen with a methoxy group at the 13-position of triptycene cause such a remarkable change in the polymer properties? Recent studies suggest that the dipole moment plays a crucial role in the self-assembly of organic molecules and polymers.^45–48^ In the 2D hexagonal arrays of 1,8,13-Trip-PDMS and 1,8-Trip-PDMS, the terminal triptycene units most likely adopt antiparallel packing to cancel their molecular dipole moments.^29^ According to density functional theory (DFT) calculations using 1,8,13-trimethoxy triptycene and 1,8-dimethoxy triptycene as models (Tables S2 and S3†), the magnitude of the dipole moments of the former (1.893 D) and latter (1.887 D) are comparable to one another, whereas their orientations with respect to the molecular axis (*c*-axis) are different (Fig. 8a and b, blue arrows). Fig. 8c and d shows 3D models of the 2D hexagonal packing of 1,8,13-trimethoxy and 1,8-dimethoxy triptycenes with an anti-parallel orientation. The dipole moment of 1,8,13-trimethoxy triptycene can be completely negated in the 2D hexagonal array, thereby reinforcing the structural integrity. However, this does not hold true for 1,8-dimethoxy triptycene, where dipole frustrations may be caused. Thus, the 2D hexagonal array of 1,8,13-trimethoxy triptycene would be more thermodynamically stable than that of 1,8-dimethoxy triptycene.

This is clearly reflected in the large difference in their melting points and might explain why, despite numerous attempts, we have not yet succeeded in obtaining single crystals from the 1,8-disubstituted triptycene derivatives we have synthesized so far. We have begun precise molecular dynamics simulations to better understand the structural aspects of the triptycene derivatives, and the results will be reported in the future.

### Self-healing properties of 1,8,13-Trip-PDMS

**Fig. 9:**
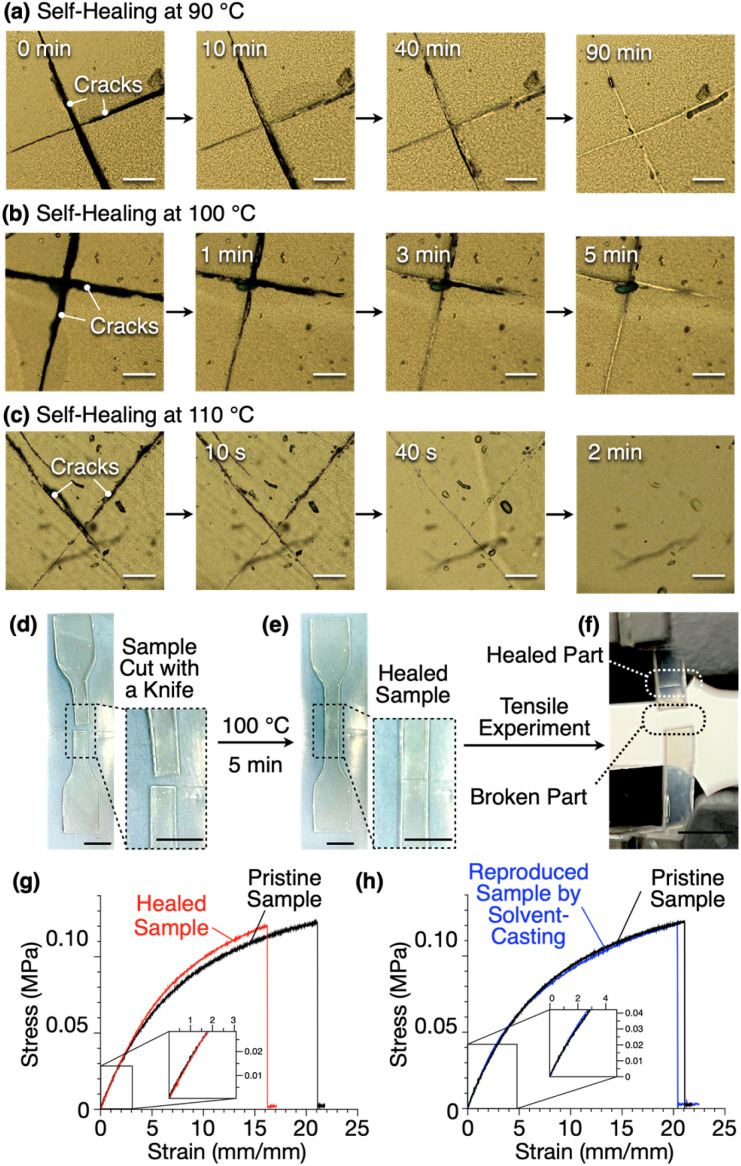
Microscopic images showing the self-healing behavior of a cracked 1,8,13-Trip-PDMS sample at (a) 90 °C, (b) 100 °C and (c) 110 °C (scale bars = 200 *μ*m). Photographs of (d) a dumbbell-shape sample of 1,8,13-Trip-PDMS after being (d) cut with a knife, (e) self-healed at 100 °C for 5 minutes and (f) a photograph of the broken sample after tensile measurement. Scale bars = 5 mm. (g) Stress–strain curves at 25 °C of pristine (black) and self-healed (red) samples of 1,8,13-Trip-PDMS and (h) of pristine (black) and reproduced (blue) samples of 1,8,13-Trip-PDMS.

Due to its non-covalently crosslinked nature, 1,8,13-Trip-PDMS exhibits self-healing and recyclability. Thus, cracks on a free-standing film of 1,8,13-Trip-PDMS healed upon heating, where the time to repair depended on the temperature; for instance, 90, 5 and 2 minutes at 90, 100 and 110 °C, respectively (Fig. 9a–c). The higher the temperature, the larger the movement of the polymer chains, thereby promoting faster reconstruction of the 2D triptycene arrays, so that the self-healing is completed in a shorter time.

We performed tensile measurements to test the mechanical properties of a film before and after self-healing (Fig. 9d–g). A dumbbell-shape film sample was prepared by punching a free-standing film prepared by casting a chloroform solution of 1,8,13-Trip-PDMS (100 mg mL^−1^) onto a Teflon sheet. The sample was cut by a knife (Fig. 9d), healed at 100 °C for 5 minutes (Fig. 9e), and then subjected to tensile tests.

The pristine sample showed a Young's modulus of 1.75 MPa, breaking strength of 0.12 MPa, and breaking elongation of 21% (Fig. 9g, black curve). After healing, these parameters were determined to be 1.90 MPa, 0.12 MPa and 16%, respectively (Fig. 9g, red curve). Obviously, the mechanical properties of the film are almost fully recovered after healing. Note that the broken part of the sample after the tensile measurement is different from the healed part (Fig. 9f), indicating an excellent self-healing ability. When a damaged sample was dissolved again in chloroform, and a dumbbell-shape sample was reproduced, the resulting sample displayed stress–strain curves (Fig. 9h, blue curve) almost identical to those observed for a pristine sample (Fig. 9h, black curve).

## Conclusions

We have presented how a seemingly slight chemical modification of a macromolecular entity can lead to surprisingly large changes in structural and physical properties. In fact, the weight fraction of methoxy groups introduced into the triptycene termini is only ∼0.3 wt% with respect to the weight of the entire PDMS molecule (1,8,13-Trip-PDMS). Moreover, unlike multiple hydrogen bonding motifs, the methoxy group, and even the triptycene unit itself, is not expected to exhibit any strong intermolecular interactions. The fact that the 1,8,13-substituted triptycene (1,8,13-Trip) attached to both termini of PDMS can make the inherently liquid polymer a hard solid without covalent cross-linking, as well as can result in a morphology that is reminiscent of a phase-separated structure of a block copolymer, unique rheological properties such as thermoplasticity and self-healing properties, all have the same origin; the ability of the terminal triptycene unit to assemble into a well-defined 2D hexagonal array, to densely network the PDMS chains and to force the domains of the networked PDMS chains to align into a long-range 1D periodic order. From a wider perspective, even without using particular functional groups capable of exhibiting strong intermolecular interaction, if a plane, in which a large number of molecules are engaged, is created, sufficient enthalpy could be gained to overcome the entropy loss associated with the decrease in the degrees of freedom of the polymer chain, and in turn, a significant improvement in the mechanical properties of polymeric materials could be achieved. The 1,8,13-substituted triptycene motif, which functions to suppress the mobility of fluidic polymer chains by making a plane, may be applicable to other polymers, not only for improving their mechanical properties but also for controlling their assembly morphology for the use of, *e.g.*, nanopatterning.

## Experimental section

### Materials

Unless otherwise stated, all commercial reagents were used as received. Hydride-terminated polydimethylsiloxanes (H-PDMS) with *M*_n_ = 18 kDa (Product #: 482064) were purchased from Sigma Aldrich Merck. 1,8,13-Trihydroxytriptycene (1)^30^ was prepared according to previously reported procedures.

### Methods

Analytical SEC was performed at 40 °C on a TOSOH GPC-8020 system equipped with a column (Shodex LF-804), a refraction index (RI) detector and a UV detector (UV-8020), where chloroform (CHCl_3_) was used as an eluent at a flow rate of 0.40 mL min^−1^. A molecular weight calibration curve was obtained using standard polystyrenes (TSKstandard polystyrene, TOSOH). NMR spectroscopy measurements were carried out on a Bruker AVANCE-500 spectrometer (500 MHz for ^1^H and 126 MHz for ^13^C). Chemical shifts (*δ*) are expressed relative to the resonances of the residual non-deuterated solvents for ^1^H [CDCl_3_: ^1^H(*δ*) = 7.26 ppm, acetone-*d*_6_: ^1^H(*δ*) = 2.05 ppm] and ^13^C{^1^H} [CDCl_3_: ^13^C(*δ*) = 77.16 ppm, acetone-*d*_6_: ^13^C(*δ*) = 29.8 and 206.3 ppm]. Absolute values of the coupling constants are given in Hertz (Hz), regardless of their sign. Multiplicities are abbreviated as singlet (s), doublet (d), triplet (t) and multiplet (m). Infrared (IR) spectra were recorded at 25 °C on a JASCO FT/IR-6600ST Fourier-transform infrared spectrometer. High-resolution APCI-TOF mass spectrometry measurements were performed on a Bruker microTOF II mass spectrometer equipped with an atmospheric pressure chemical ionization (APCI) probe. DSC measurements were carried out on a Mettler–Toledo DSC 1 differential scanning calorimeter, where temperature and enthalpy were calibrated with in (430 K, 3.3 J mol^−1^) and Zn (692.7 K, 12 J mol^−1^) standard samples in sealed Al pans. Cooling and heating profiles were recorded and analyzed using the Mettler–Toledo STAR^e^ software system. Thermogravimetric analysis (TGA) was performed on a SHIMADZU TGA-50 analyzer. Rheological measurements were performed on an Anton Paar MCR102 rotational rheometer equipped with a parallel-plate-type jig with a diameter of 2 cm and a sample gap of 300 *μ*m. Prior to the rheological measurements, samples were heated at 200 °C for 10 min, and the data were obtained on cooling from 200 to 30 °C under a strain of 0.1%. Variable-temperature powder XRD of polymer samples were measured in a glass capillary with a diameter of 1.5 mm using a Rigaku NANOPIX equipped with a HyPix-6000 (Rigaku) detector. The scattering vector (*q* = 4π sin *θ*/*λ*), scattering angle *θ* and the position of the incident X-ray beam on the detectors were calibrated using several orders of layer reflections from silver behenate (*d* = 58.380 Å), where *λ* refers to the wavelength of the X-ray beam (Cu Kα, 1.54 Å). The sample-to-detector distance was *ca*. 90 mm. The obtained diffraction patterns were integrated along the Debye–Scherrer ring to afford 1D intensity data using the Rigaku 2DP software. The cell parameters were refined using CellCalc ver. 2.10 software. For the self-healing test, optical microscopy (OM) was performed on a Nikon Eclipse LV100POL optical polarizing microscope equipped with a Mettler–Toledo HS1 controller attached to a HS82 hot stage. Atomic force microscopy (AFM) measurements of the thin-film of 1,8,13-Trip-PDMS were performed on a Bruker Dimension Icon atomic force microscope operated in tapping mode using a silicon cantilever tip (OMCL-AC160TS, Olympus Corp., Japan) with a nominal tip radius of 7 nm. Experiment data were obtained by a NanoScope V controller with a NanoScope software 9.7, and further analyzed using NanoScope Analysis 2.0 software. The thin-film sample of 1,8,13-Trip-PDMS for AFM measurement was prepared by spin-coating (1000 rpm, 2 min) on the silicon wafer from THF solution (10 mg mL^−1^), heated at 200 °C under vacuum, and then cooled at a rate of 1 °C min^−1^. Tensile measurement was carried out using an INSTRON universal testing machine (6800 Single Column Table Model) with a 250 N load cell at a strain rate of 0.5 mm min^−1^ at 25 °C. Density functional theory (DFT) calculations were performed using the Gaussian 16 program package.^49^ Geometry optimization and calculation of the dipole moment were performed at the B3LYP/6-31G(d) level of calculations. The Cartesian coordinates and energy of the optimized structure are listed in Tables S2 and S3.†

## Data availability

All experimental data associated with this work are available in the ESI.†

## Author contributions

F. I. and T. F. conceive the project; F. I., To. F. and T. F. designed the experiments; Y. C. and F. I. carried out the synthesis and characterization of the materials; Y. C., F. I. and T. K. performed the X-ray diffraction experiments and analysed the data; Y. C., To. F., H. L., X. L. and K. N. performed the AFM measurement and analysed the data; Y. C., F. I., To. F. and M. T. performed the rheological measurement and analysed the data; Y. C. and To. F. performed the tensile measurement and analysed the data; Y. C., F. I., To. F. and T. F. co-wrote the manuscript.

## Conflicts of interest

There are no conflicts to declare.

## Supplementary Material

SC-014-D2SC05491D-s001
