# Supplementary material for: Overcoming the entropy of polymer chains by making a plane with terminal groups: a thermoplastic PDMS with a long-range 1D structural order
Source: Chem Sci. 2023 Jan 4;14(9):2431–40. doi: 10.1039/d2sc05491d (PMC9977418; doi:10.1039/d2sc05491d)
Supplement: SC-014-D2SC05491D-s001 [file SC-014-D2SC05491D-s001.pdf]

Electronic Supplementary Material (ESI) for Chemical Science.
This journal is © The Royal Society of Chemistry 2023

Supplementary Information

Overcoming the entropy of polymer chains by making a plane with terminal groups: A thermoplastic PDMS with a long-range 1D structural order

Yugen Chen, Fumitaka Ishiwari*, Tomoya Fukui, Takashi Kajitani, Haonan Liu, Xiaobin Liang, Ken Nakajima, Masatoshi Tokita, Takanori Fukushima*

*To whom correspondence should be addressed.
E-mail: ishiwari@chem.eng.osaka-u.ac.jp; fukusihma@res.titech.ac.jp

Table of Contents

1. Synthesis (Scheme S1) ··· S2
2. Supporting Figures (Figs. S1–S23) ··· S5
3. Supporting Tables (Tables S1–S3) ··· S19
4. Supporting References ··· S22

1. Synthesis

Scheme S1. Synthesis of 1,8,13-Trip-PDMS.

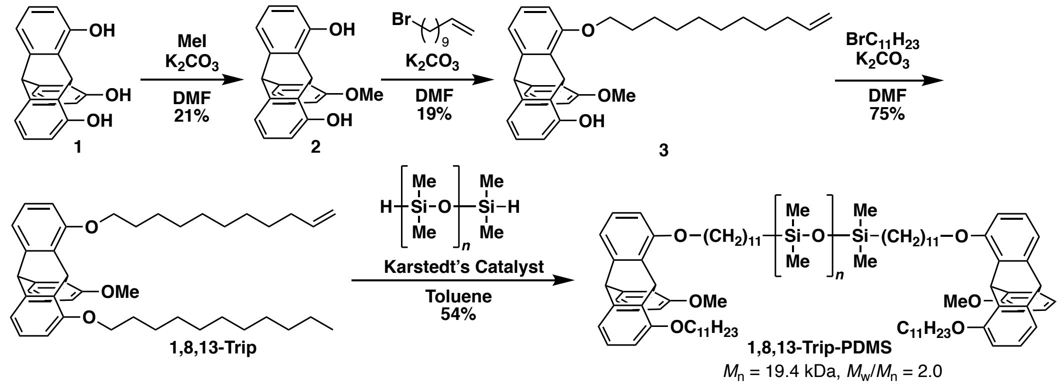

1,8-Dihydroxyl-13-methoxytriptycene (2). Under nitrogen, iodomethane (1.08 mL, 17.4 mmol) was added at 25 °C to a *N,N*-dimethylformamide (DMF) solution (70 mL) of a mixture of 1,8,13-trihydoroxytriptycene[S1] (3.50 g, 11.6 mmol) and potassium carbonate (4.80 g, 34.8 mmol), and the resulting mixture was stirred at 75 °C for 18 h. After being allowed to cool to 25 °C, the reaction mixture was poured into water and extracted with diethyl ether. A combined organic extract was washed with brine, dried over anhydrous magnesium sulfate, and then evaporated to dryness under reduced pressure. The residue was subjected to column chromatography on silica (chloroform/acetone 7/3 v/v) to allow isolation of **2** as a white solid (770 mg, 2.43 mmol) in 21% yield: ^{1}H NMR (500 MHz, 298 K, acetone-d_6): δ (ppm) 8.29 (s, 2H), 7.05 (d, J = 7.3 Hz, 1H), 6.94–6.90 (m, 3H), 6.88 (s, 1H), 6.77 (dd, J = 7.3, 8.1 Hz, 2H), 6.66 (dd, J = 8.1, 0.9 Hz, 1H), 6.55 (dd, J = 8.1, 0.9 Hz, 2H), 5.47 (s, 1H), 3.84 (s, 3H). ^{13}C{^{1}H} NMR (126 MHz, 298 K, acetone-d_6): δ (ppm) 155.4, 152.7, 152.6, 149.8, 149.7, 134.5, 132.2, 126.4, 126.1, 117.3, 116.2, 113.6, 109.4, 56.1, 55.4, 34.4. FT-IR (KBr): ν (cm^{-1}) 3297, 2954, 2838, 1598, 1475, 1454, 1442, 1360, 1315, 1278, 1229, 1192, 1161, 1086, 1066, 1018, 785, 724. APCI-TOF-mass: calcd. for $C_{21}H_{16}O_3$ [M]$^+$; m/z = 316.1094; found: 316.1046. ^{1}H and ^{13}C{^{1}H} NMR, FT-IR, and high-resolution APCI mass spectra of **2** are shown in Figs. S2, S3, S4, and S5, respectively.

1-(10-Undecenoxy)-8-hydroxyl-13-methoxytriptycene (3). Under nitrogen, 11-bromo-1-undecene (0.230 mL, 1.11 mmol) was added at 25 °C to a *N,N*-dimethylformamide (DMF) solution (12 mL) of a mixture of 1,8-dihydroxyl-13-methoxytriptycene (**2**) (512 mg, 1.62 mmol) and potassium carbonate (670 mg, 4.86 mmol), and the resulting mixture was stirred at 75 °C for 18 h. After being allowed to cool to 25 °C, the reaction mixture was poured into water and extracted with diethyl ether. A combined organic extract was washed with brine, dried over anhydrous magnesium sulfate, and then evaporated to dryness under reduced pressure. The residue was subjected to column chromatography on silica (chloroform) to allow isolation of **3** (racemic mixture) as a white solid (98 mg, 0.21 mmol) in 19% yield. ^{1}H NMR (500 MHz, $CDCl_3$): δ (ppm) 7.03–6.98 (m, 3H), 6.93 (dd, J = 7.8, 7.8 Hz, 1H), 6.90 (dd, J = 7.8, 7.8 Hz,

1H), 6.83 (dd, J = 7.8, 7.8 Hz, 1H), 6.67 (s, 1H), 6.59 (d, J = 8.2 Hz, 1H), 6.57 (d, J = 8.2 Hz, 1H), 6.51 (dd, J = 8.2, 0.7 Hz, 1H), 5.82 (ddt, J = 17.0, 10.3, 6.7 Hz, 1H), 5.38 (s, 1H), 5.17 (s, 1H), 5.00 (dd, J = 17.1, 1.9 Hz, 1H), 4.94 (dd, J = 10.2, 1.0 Hz, 1H), 4.04–3.96 (m, 2H), 3.86 (s, 1H) 2.05 (dt, J = 7.2, 6.9 Hz, 2H), 1.91–1.79 (m, 2H), 1.58–1.48 (m, 2H), 1.40–1.26 (m, 10H). $^{13}C\{^1H\}$ NMR (126 MHz, $CDCl_3$): δ (ppm) 154.5, 153.9, 150.3, 148.7, 148.4, 148.3, 139.4, 133.3, 132.9, 130.8, 126.0, 125.9, 125.9, 116.7, 116.7, 116.7, 114.3, 113.4, 110.3, 108.9, 69.1, 62.4, 56.1, 54.7, 34.0, 33.2, 29.8, 29.6, 29.3, 29.1, 26.2. FT-IR (KBr): v (cm^{-1}) 2923, 2841, 1592, 1518, 1465, 1273, 1087, 1057, 903, 788, 729. APCI-TOF-mass: calcd. for $C_{32}H_{36}O_3$ $[M]^+$; m/z = 468.2664; found: 468.2421. ^1H and $^{13}C\{^1H\}$ NMR, FT-IR, and high-resolution APCI mass spectra of **3** (racemic mixture) are shown in Figs. S6, S7, S8, and S9, respectively.

1-(10-Undecenoxy)-8-undecyloxy-13-methoxytriptycene (1,8,13-Trip). Under nitrogen, 1-bromoundecane (0.068 mL, 0.28 mmol) was added at 25 °C to a *N,N*-dimethylformamide (DMF) solution (2 mL) of a mixture of 1-(10-undecenoxy)-8-hydroxyl-13-methoxytriptycene (**3**) (58 mg, 0.12 mmol) and potassium carbonate (50 mg, 0.36 mmol), and the resulting mixture was stirred at 75 °C for 18 h. After being allowed to cool to 25 °C, the reaction mixture was poured into water and extracted with diethyl ether. A combined organic extract was washed with brine, dried over anhydrous magnesium sulfate and then evaporated to dryness under reduced pressure. The residue was subjected to column chromatography on silica (chloroform/hexane 1/2 v/v) to allow isolation of 1,8,13-Trip (racemic mixture) as a white solid (59 mg, 0.09 mmol) in 75% yield. ^1H NMR (500 MHz, $CDCl_3$): δ (ppm) 7.03–7.00 (m, 3H), 6.91 (dd, J = 7.7, 7.7 Hz, 1H), 6.89 (s, 1H), 6.88 (dd, J = 7.7, 7.7 Hz, 2H), 6.59–6.56 (m, 3H), 5.84 (ddt, J = 17.0, 10.3, 6.7 Hz, 1H), 5.39 (s, 1H), 5.02 (dd, J = 17.0, 1.6 Hz, 1H), 4.95 (dd, J = 10.1, 0.9 Hz, 1H), 4.03–3.94 (m, 4H), 3.85 (s, 3H), 2.07 (dt, J = 7.1, 7.0 Hz, 2H), 1.93–1.80 (m, 4H), 1.67–1.53 (m, 4H), 1.45–1.24 (m, 24H), 0.91 (t, J = 6.7 Hz, 3H). $^{13}C\{^1H\}$ NMR (126 MHz, $CDCl_3$): δ (ppm) 154.8, 154.2, 148.8, 148.6, 139.4, 134.0, 133.7, 125.7, 125.6, 116.7, 116.7, 114.3, 110.7, 109.2, 69.2, 69.2, 56.3, 54.7, 34.0, 33.7, 32.1, 29.9, 29.9, 29.8, 29.8, 29.7, 29.6, 29.4, 29.1, 26.2, 26.2, 22.9, 14.3. FT-IR (KBr): v (cm^{-1}) 2923, 2851, 1596, 1484, 1466, 1433, 1277, 1100, 1062, 905, 787, 729. APCI-TOF-mass: calcd. for $C_{43}H_{58}O_2$ $[M]^+$; m/z = 622.4380; found: 622.4274. ^1H and $^{13}C\{^1H\}$ NMR, FT-IR, and high-resolution APCI mass spectra of 1,8,13-Trip (racemic mixture) are shown in Figs. S10, S11, S12, and S13, respectively.

1,8,13-Trip-PDMS. Under nitrogen, a xylene solution of Karstedt's catalyst (Pt ~2%, 12.3 μL) was added at 25 °C to a toluene solution (2 mL) of a mixture of 1,8,13-Trip (racemic mixture, 60 mg, 96.4 μmol) and H-PDMS (M_n = 18 kDa, 0.34 mL, 0.024 mmol). The resulting mixture was stirred at 115 °C for 24 h and then allowed to cool to 25 °C. Methanol was added to the reaction mixture, and a lower liquid layer separated was corrected by decantation. The residue was subjected to column chromatography on silica (ethyl acetate) to allow separation of a fraction containing 1,8,13-Trip-PDMS, which was further purified by preparative scale SEC to give 1,8,13-Trip-PDMS (M_n = 19.4 kDa, 245.2 mg, 0.013 mmol) as a colorless solid in 54%

yield: ^{1}H NMR (500 MHz, 298 K, $CDCl_3$): δ (ppm) 7.01–6.99 (m, 6H), 6.91–6.86 (m, 8H), 6.58–6.55 (m, 6H), 5.37 (s, 2H), 4.02–3.93 (m, 8H), 3.84 (s, 6H), 1.90–1.80 (m, 8H), 1.65–1.52 (m, 8H), 1.44–1.20 (m, 56H), 0.89 (t, J = 6.4 Hz, 6H), 0.53 (t, J = 6.9 Hz, 4H), 0.18 – –0.05 (m, 1473H). FT-IR (KBr): ν (cm^{-1}) 2950, 1258, 1080, 1014, 789, 687. M_n (NMR) = 19,439 Da, M_n (SEC) = 14,680 Da, M_w/M_n (SEC) = 2.0. ^{1}H NMR spectrum, FT-IR spectrum, and SEC chart of 1,8,13-TripPDMS are shown in Figs. S16, S17, and S18, respectively.

2. Supporting Figures

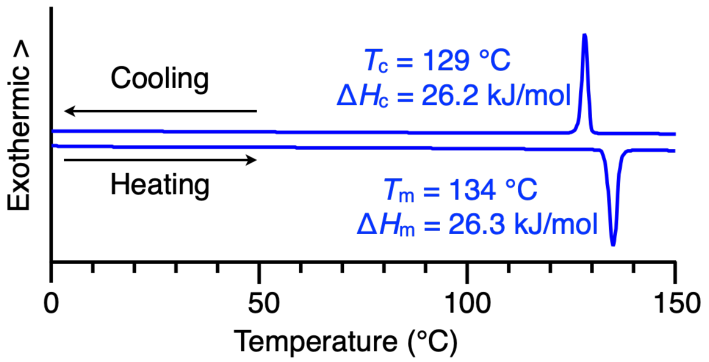

Fig. S1 DSC profile of 1,8-bis(dodecyloxy)triptycene[S2] in a second heating/cooling cycle, measured at a scan rate of 10 °C/min under N_2 flow (50 mL/min).

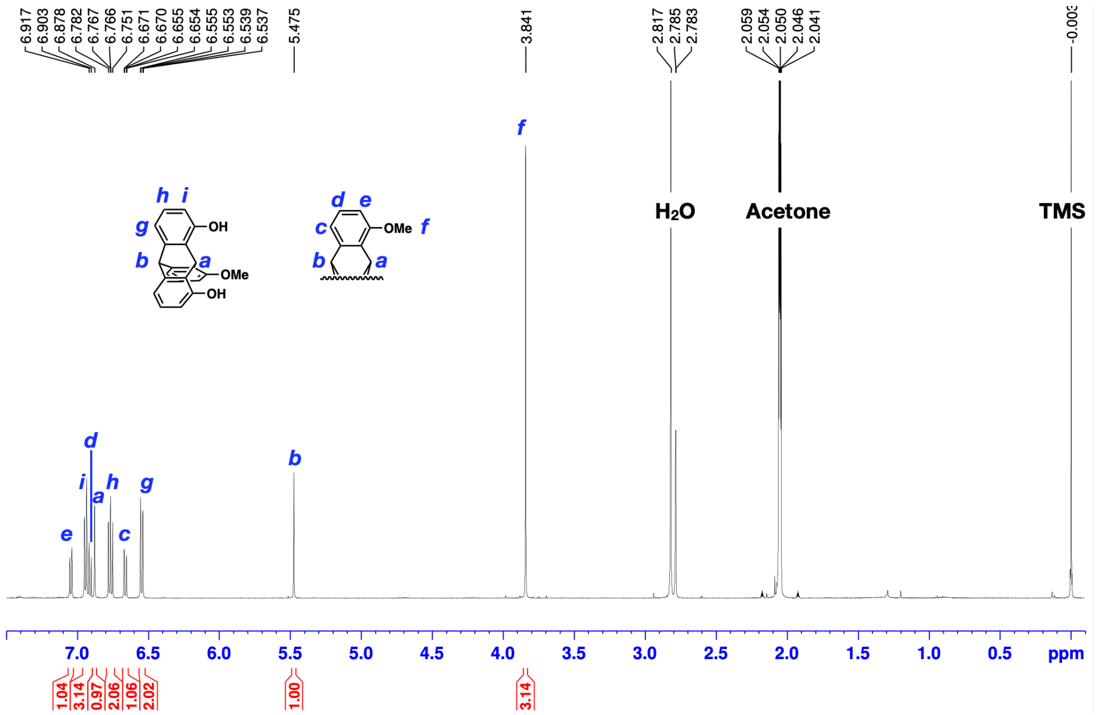

Fig. S2 ^{1}H NMR spectrum (500 MHz) of **2** in acetone-d_6 at 25 °C.

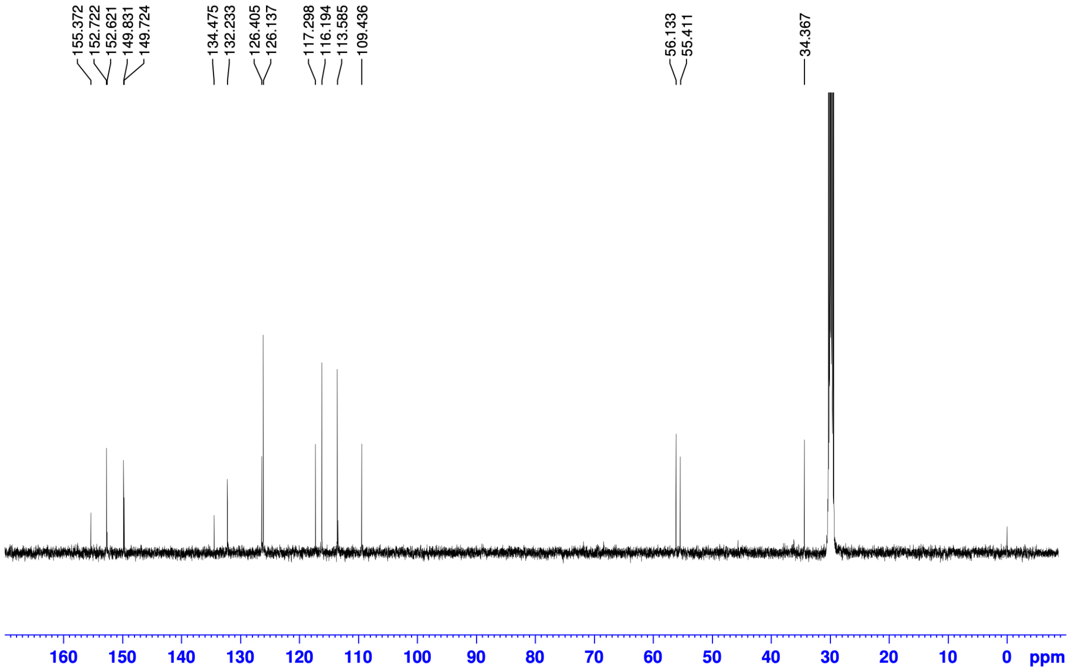

Fig. S3 $^{13}C\{^{1}H\}$ NMR spectrum (126 MHz) of **2** in acetone-d_6 at 25 °C.

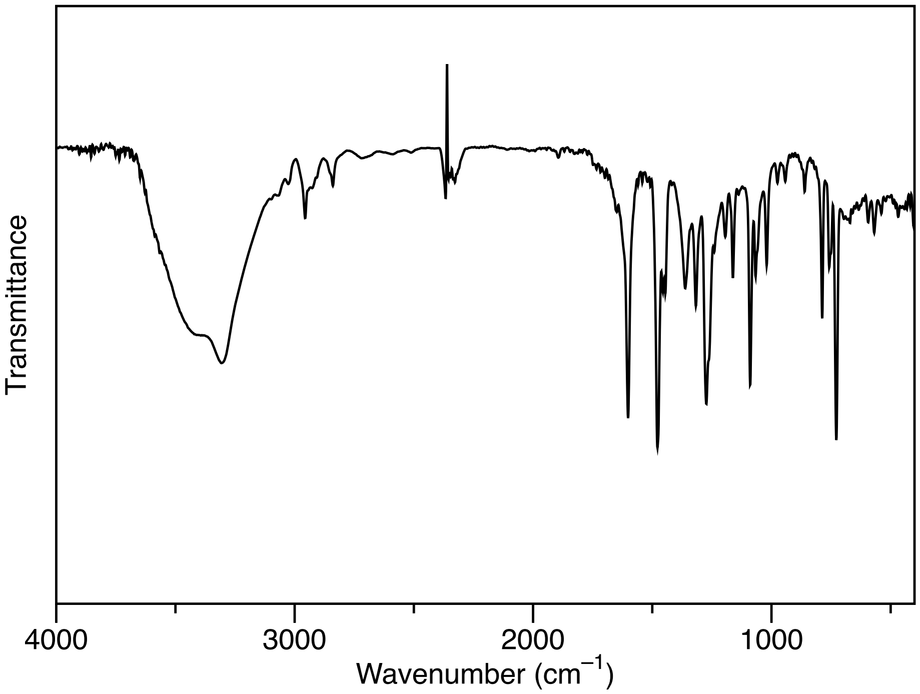

Fig. S4 FT-IR spectrum of **2** at 25 °C (KBr).

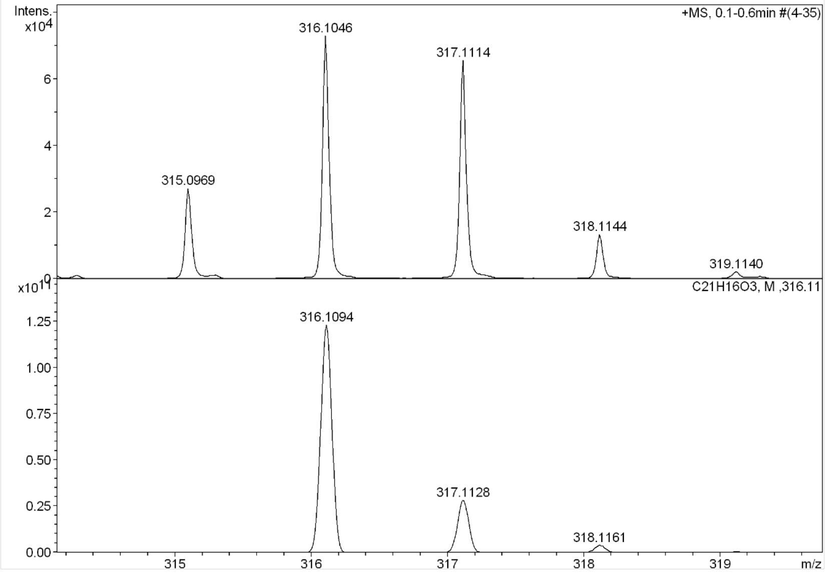

Fig. S5 Observed (upper) and simulated (lower) high-resolution APCI mass spectra of **2**.

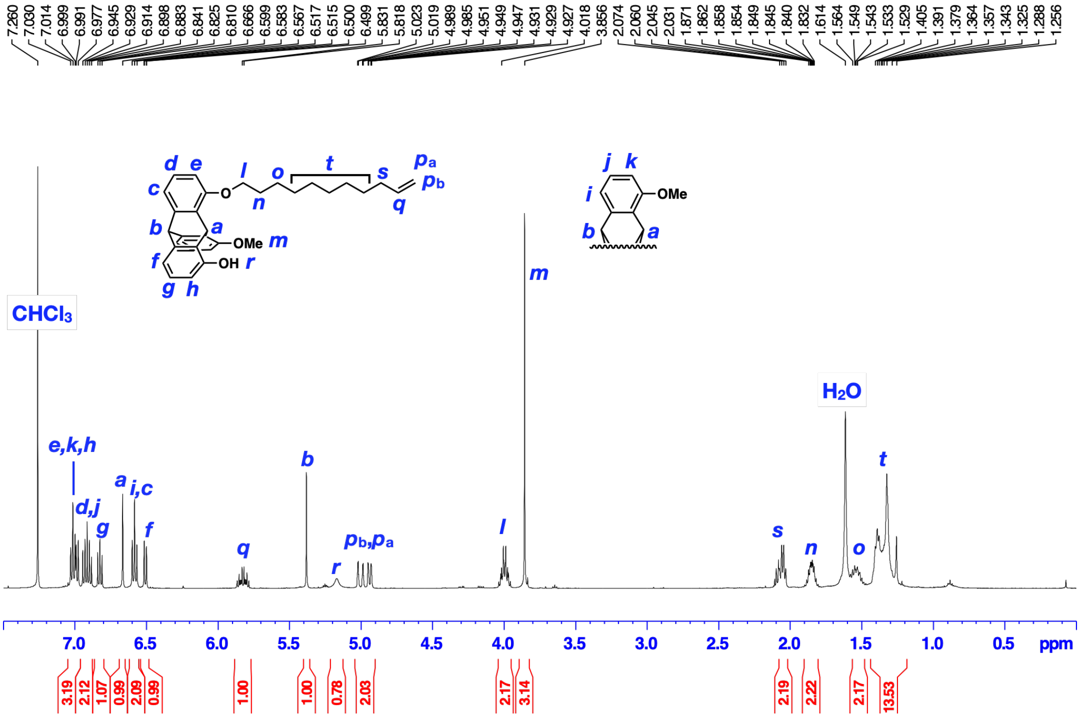

Fig. S6 ^{1}H NMR spectrum (500 MHz) of **3** in $CDCl_3$ at 25 °C.

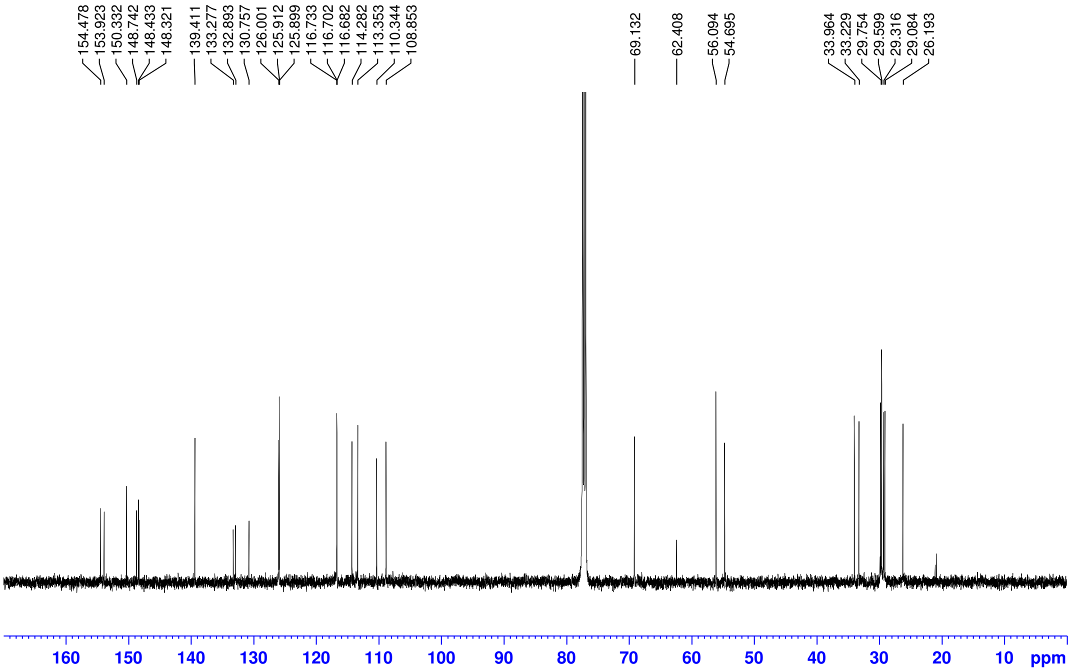

Fig. S7 $^{13}C\{^{1}H\}$ NMR spectrum (126 MHz) of **3** in $CDCl_3$ at 25 °C.

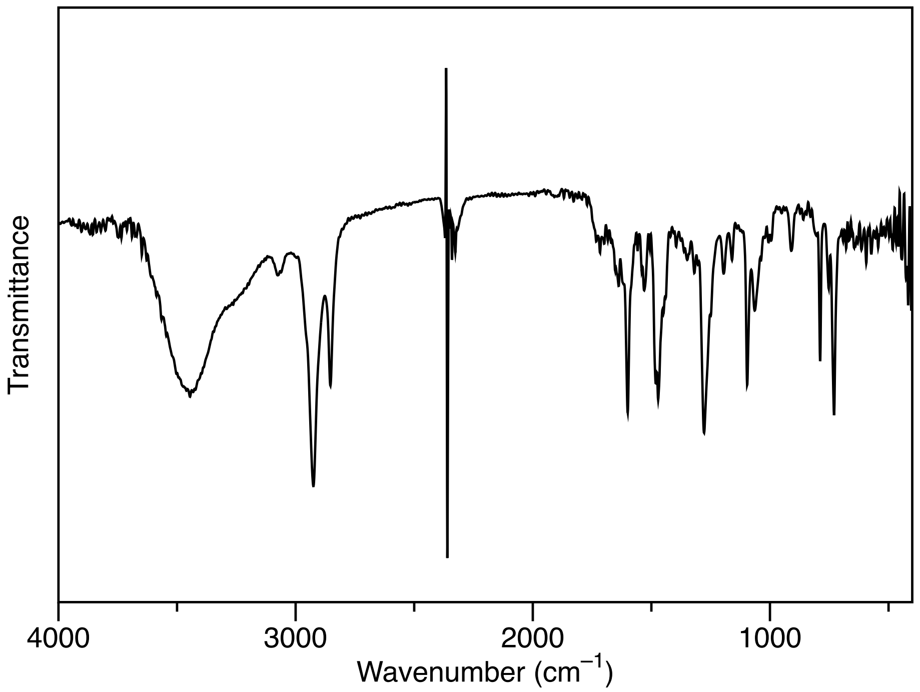

Fig. S8 FT-IR spectrum of **3** at 25 °C (KBr).

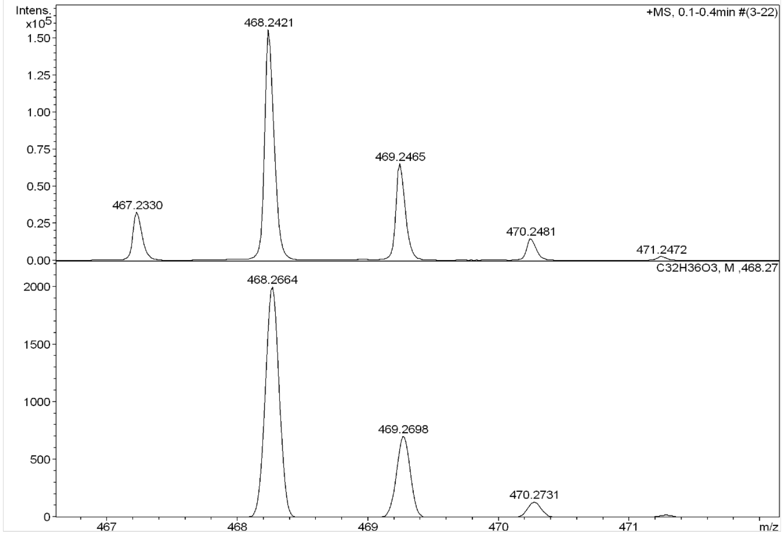

Fig. S9 Observed (upper) and simulated (lower) high-resolution APCI mass spectra of **3**.

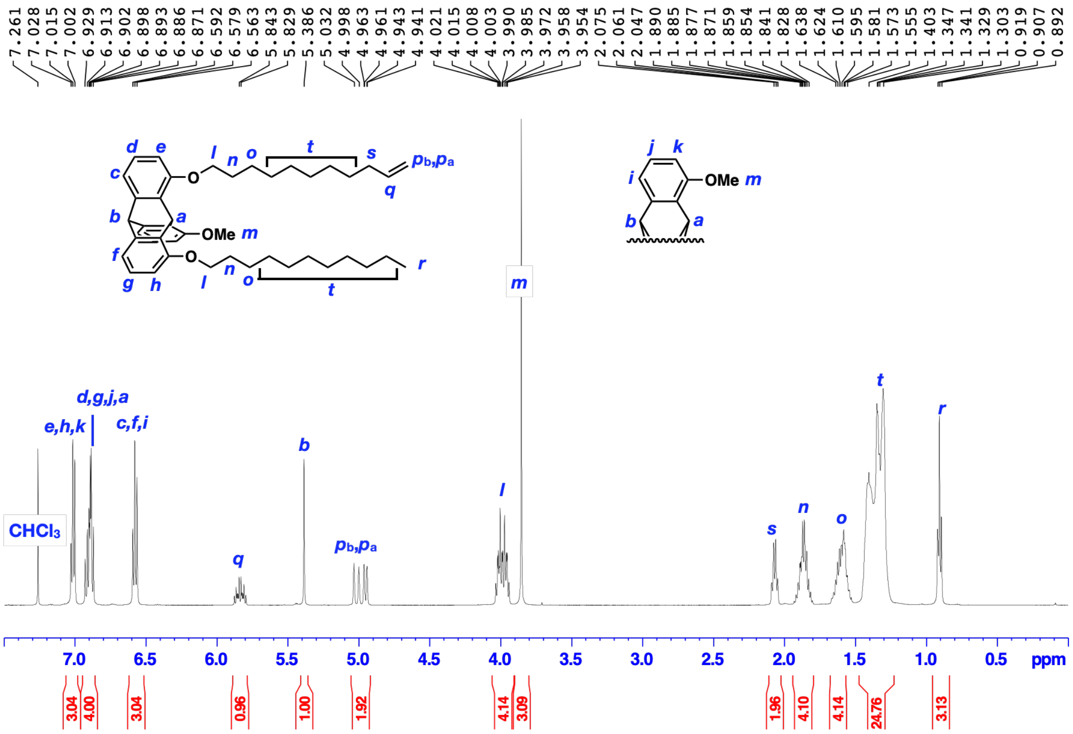

Fig. S10 ^{1}H NMR spectrum (500 MHz) of 1,8,13-Trip in $CDCl_3$ at 25 °C.

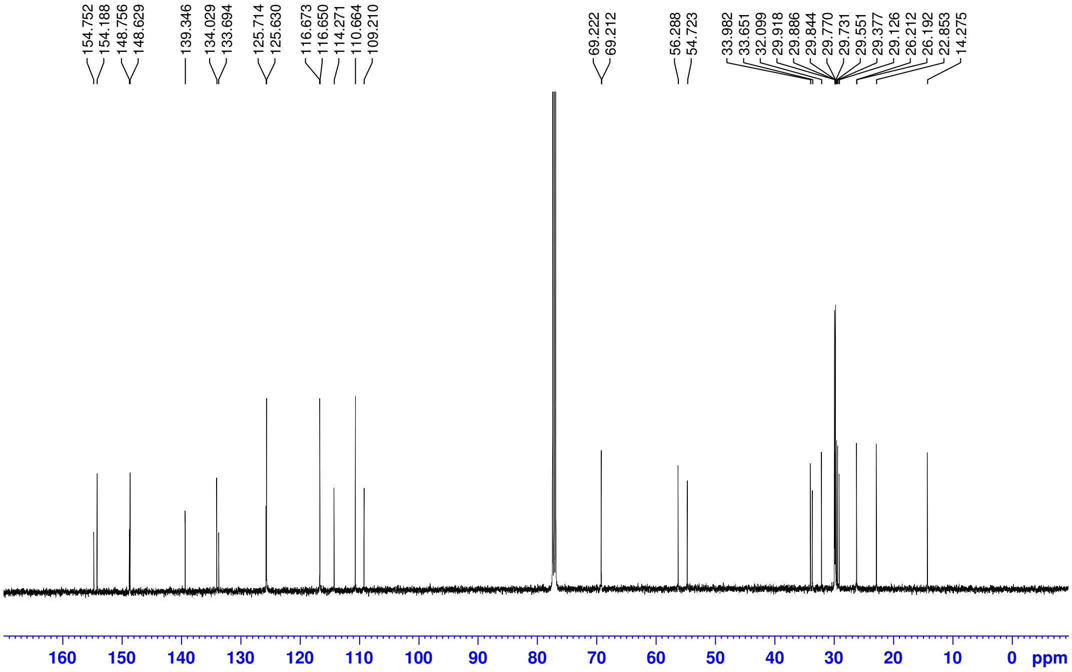

Fig. S11 $^{13}C\{^{1}H\}$ NMR spectrum (126 MHz) of 1,8,13-Trip in $CDCl_3$ at 25 °C.

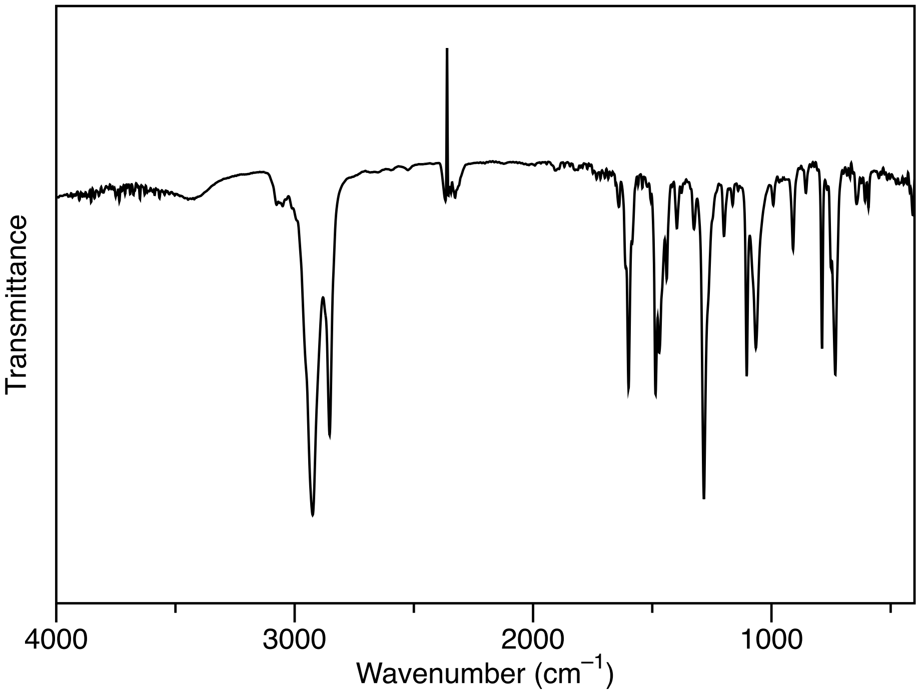

Fig. S12 FT-IR spectrum of 1,8,13-Trip at 25 °C (KBr).

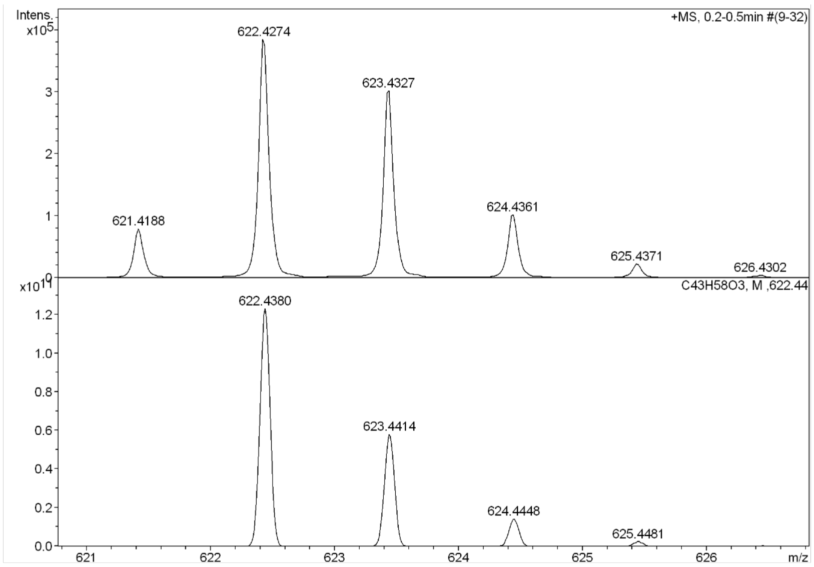

Fig. S13 Observed (upper) and simulated (lower) high-resolution APCI mass spectra of 1,8,13-Trip.

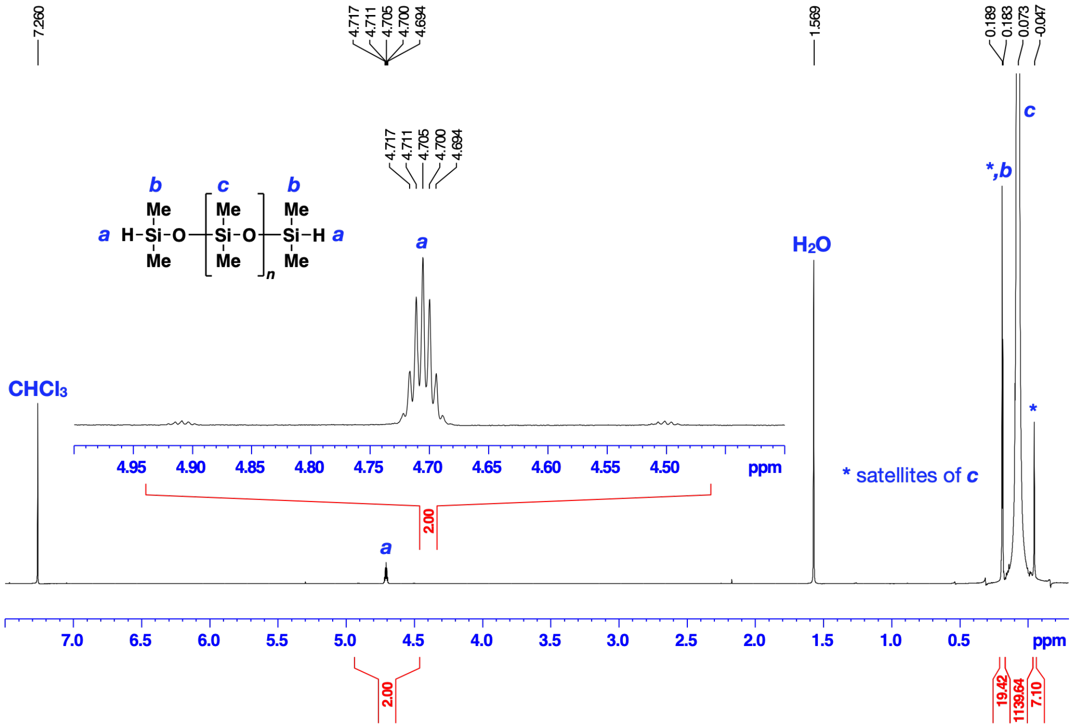

Fig. S14 ^{1}H NMR spectrum (500 MHz) of H-PDMS in $CDCl_3$ at 25 °C.

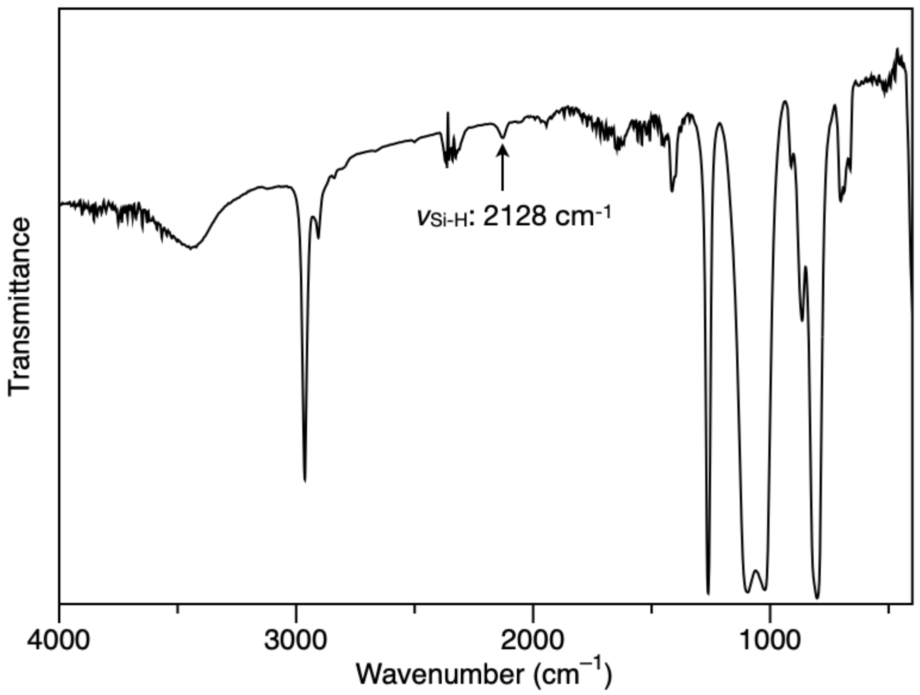

Fig. S15 FT-IR spectrum of H-PDMS at 25 °C (KBr).

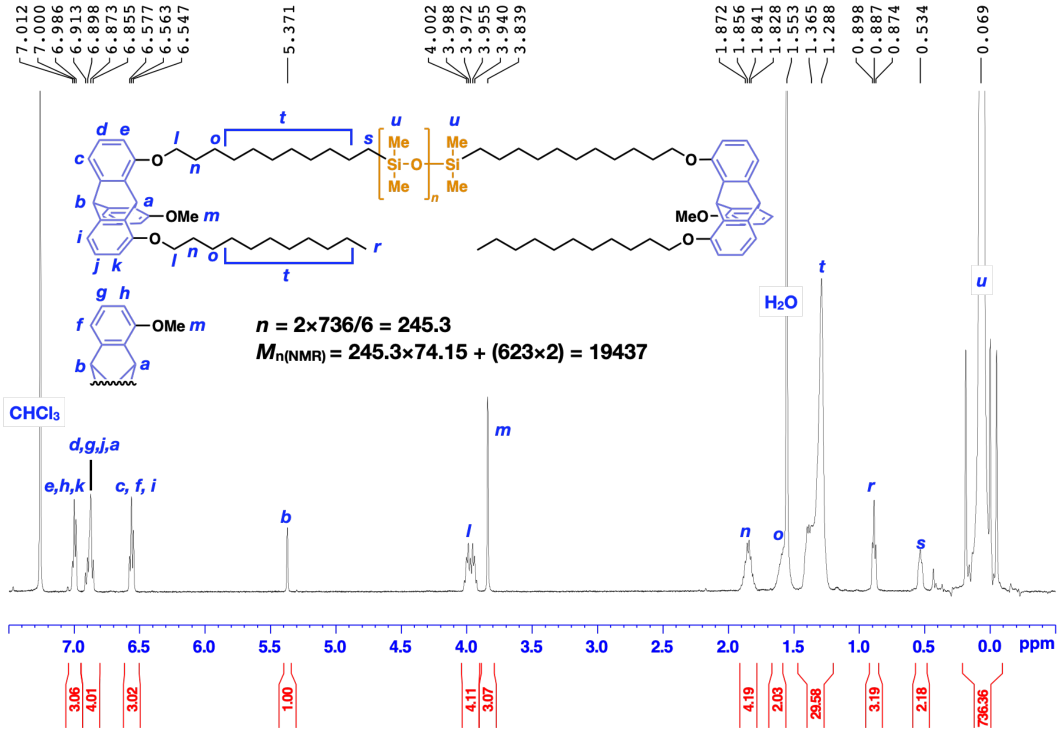

Fig. S16 ^{1}H NMR spectrum (500 MHz) of 1,8,13-Trip-PDMS in $CDCl_3$ at 25 °C.

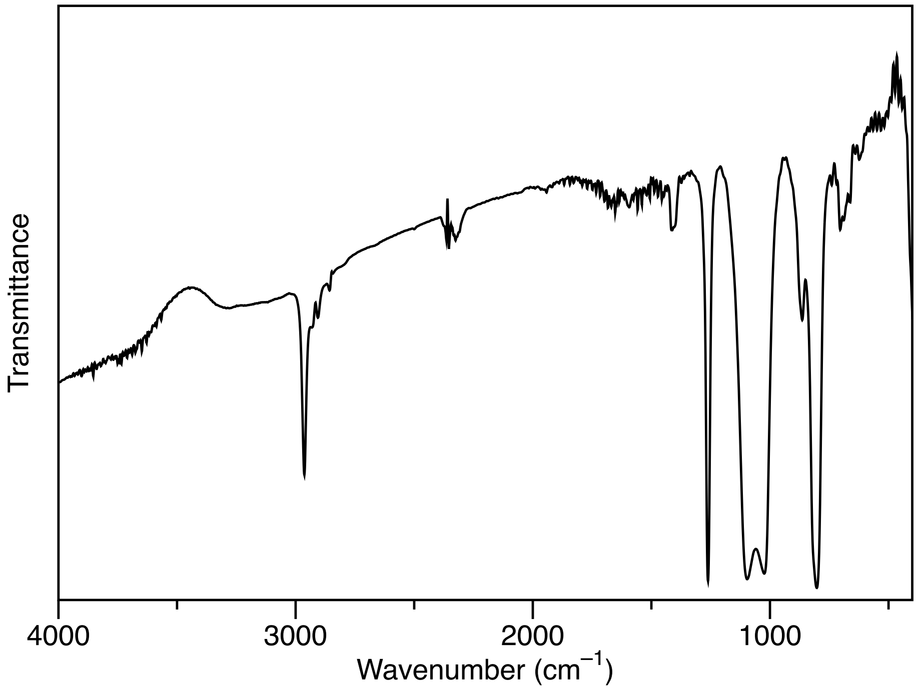

Fig. S17 FT-IR spectrum of 1,8,13-Trip-PDMS at 25 °C (KBr).

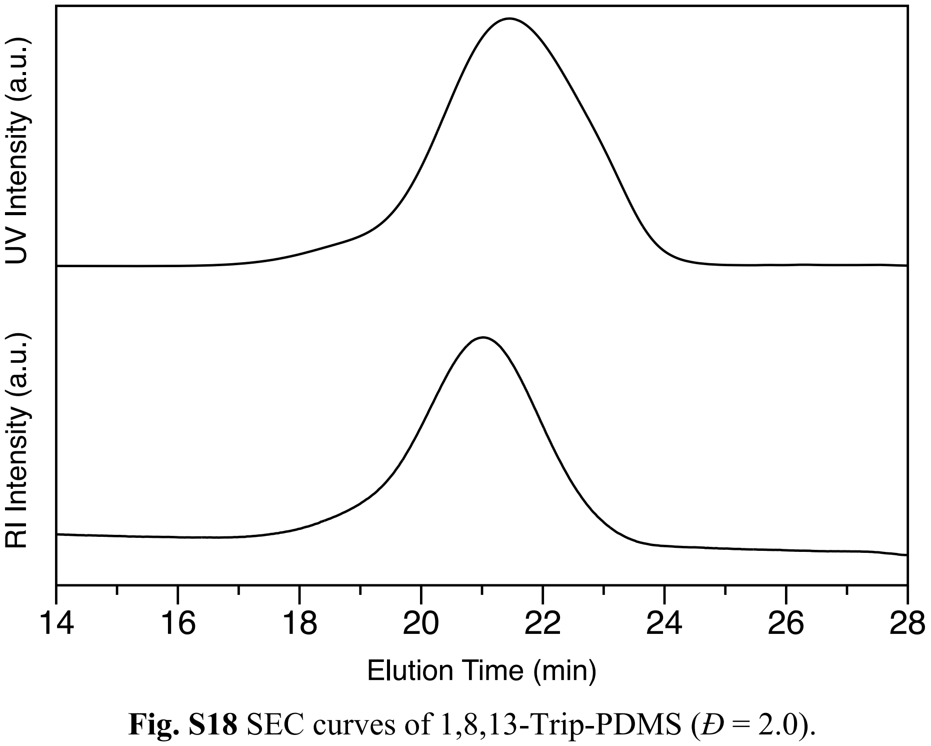

Fig. S18 SEC curves of 1,8,13-Trip-PDMS (*Đ* = 2.0).

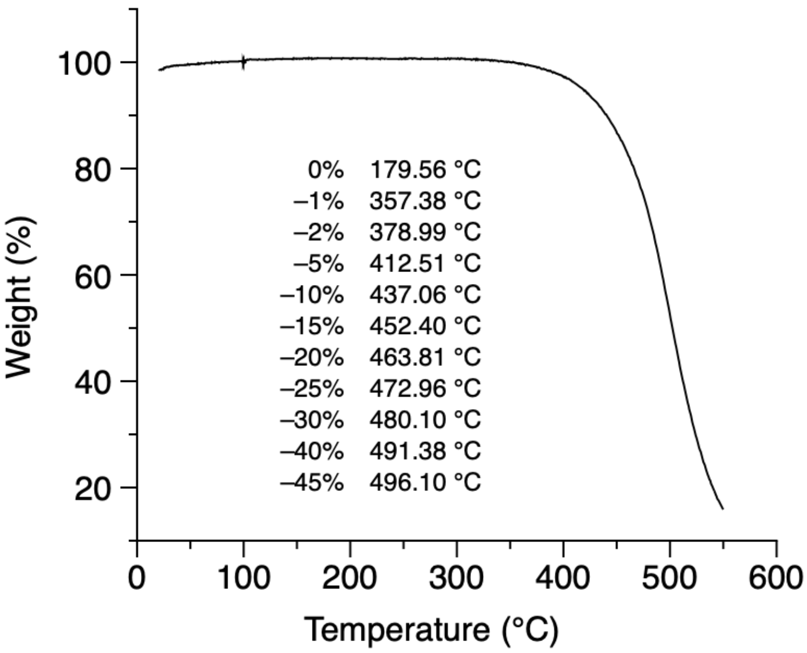

Fig. S19 TGA profile of 1,8,13-Trip-PDMS measured at a scan rate of 10 °C/min under N_2 flow (50 mL/min).

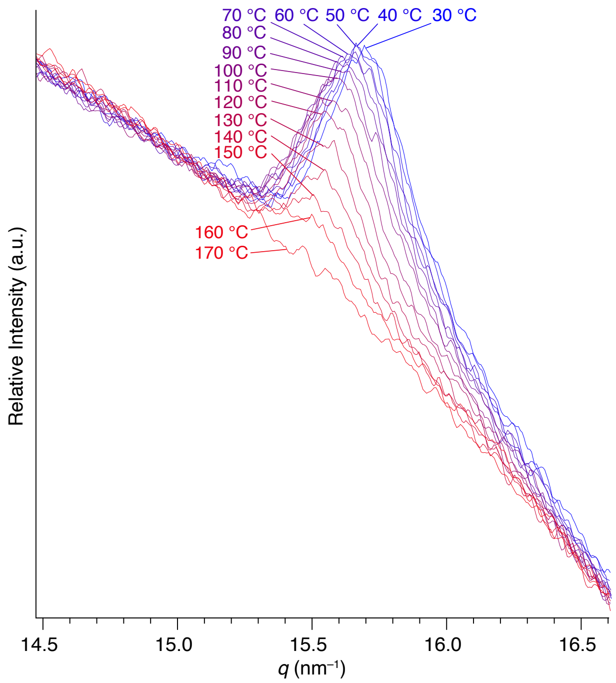

Fig. S20 Temperature-dependence of the intensity of XRD diffraction from the (110) plane of 1,8,13-Trip-PDMS (magnified profiles of the powder XRD patterns shown in Fig. 5a)

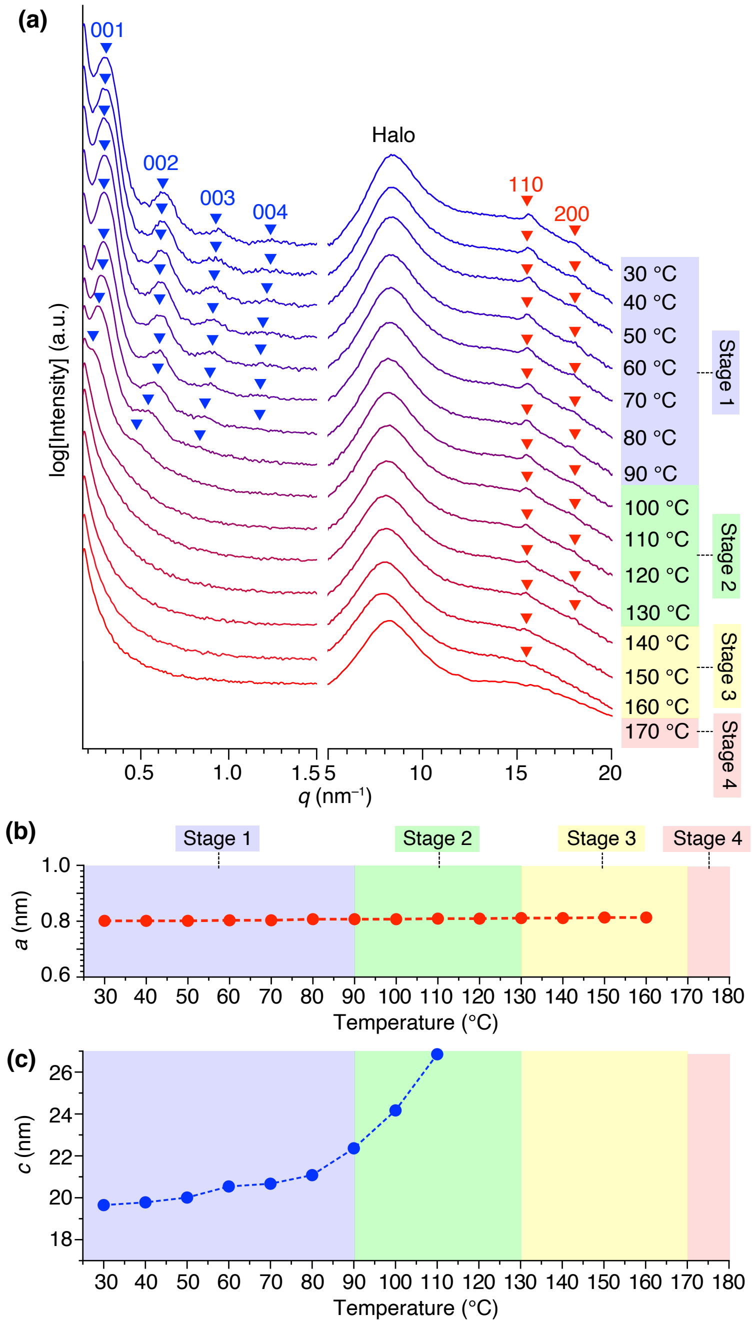

Fig. S21 (a) Variable-temperature small- and wide-angle XRD patterns of 1,8,13-Trip-PDMS measured upon cooling in a glass capillary with a diameter of 1.5 mm. Right panels represent the temperature ranges of Stages 1–4 in Fig. 2b. Temperature-dependence of (b) 2D hexagonal lattice parameter and (c) 1D layer spacing.

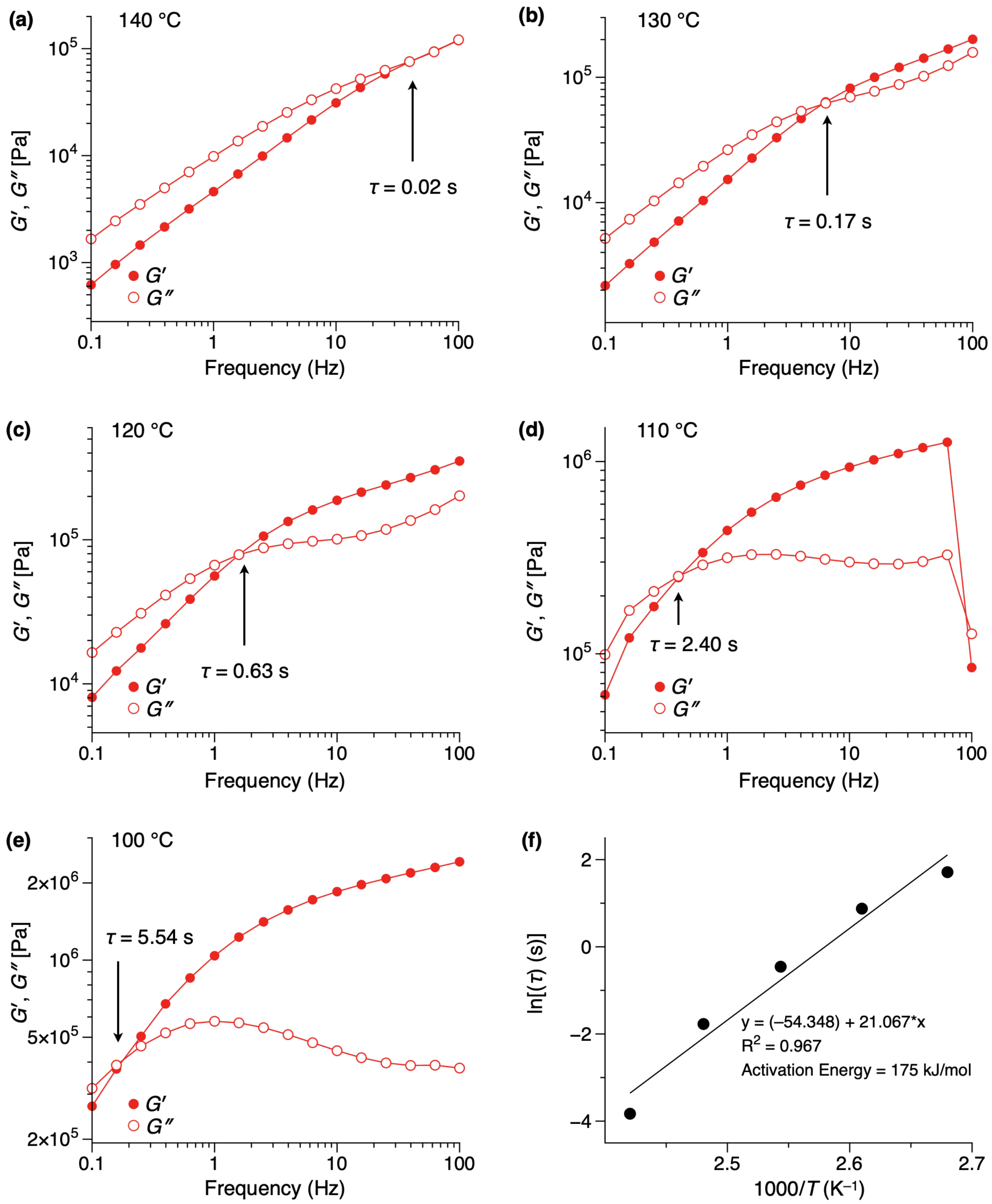

Fig. S22 Frequency-dependence of the storage modulus (*G*′, filled circle) and loss modulus (*G*″, open circle) of 1,8,13-Trip-PDMS at (a) 140, (b) 130, (c) 120, (d) 110 and (e) 100 °C. (f) Arrhenius plot of the relaxation time versus reciprocal of the measurement temperature. The activation energy for relaxation of 1,8,13-Trip-PDMS was estimated to be 175 kJ/mol.

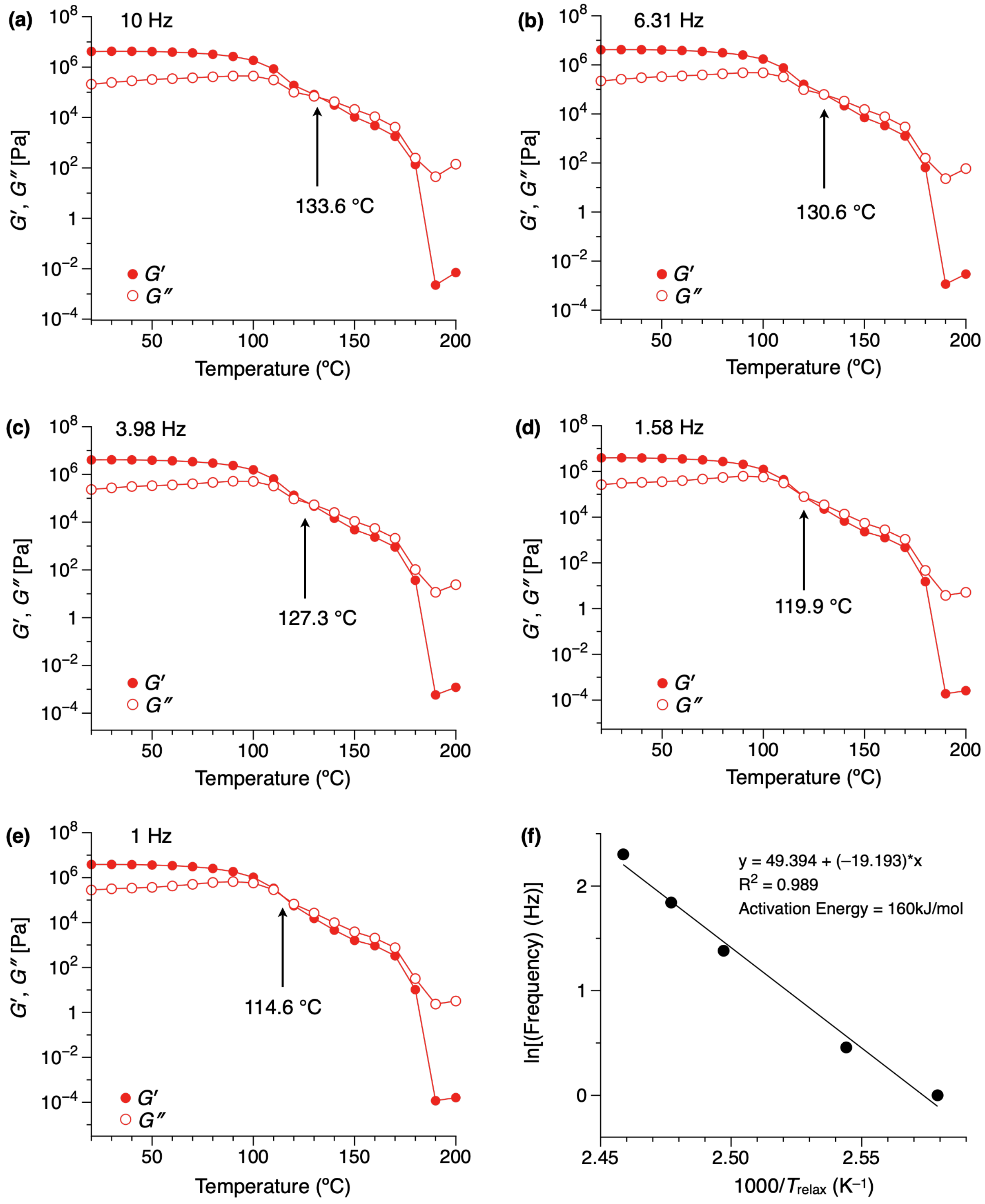

Fig. S23 Temperature-dependence of the storage modulus (G', filled circle) and loss modulus (G'', open circle) of 1,8,13-Trip-PDMS at a measurement frequency of (a) 10, (b) 6.31, (c) 3.98, (d) 1.58, and (e) 1.00 Hz. (f) Arrhenius plot of the measurement frequency versus $1/T_{relax}$. The activation energy for relaxation of 1,8,13-Trip-PDMS was estimated to be 160 kJ/mol.

3. Supporting Tables

Table S1. Peak assignments of the powder XRD pattern (Fig. 3a) of 1,8,13-Trip at 25 °C.

Temp. (°C)	q (nm^{-1})	$d_{obs.}$ (nm)	$d_{calc.}$ (nm)	*hkl*
25	3.510	1.790	1.793	001
(*P*6*mm*)[a]	7.020	0.895	0.897	002
	9.109	0.690	0.691	100
	9.661	0.650	0.649	101
	10.530	0.597	0.598	003
	11.426	0.550	0.550	102
	14.032	0.448	0.448	004
	15.712	0.400	0.402	110
	16.0299	0.392	0.392	111
	18.122	0.347	0.348	200
	18.367	0.342	0.342	201

[a]Hexagonal lattice parameters (a) = 0.800 nm and (c) = 1.790 nm.

Table S2. Cartesian coordinates (in Å) of the optimized structure of 1,8,13-trimethoxy-triptycene [RωB97X-D/6-31G(d)][S3] (Fig. 8a). The Gaussian16 software was used for the DFT calculations.[S4]

O	2.436039	0.573880	1.906843	C	3.596246	0.845740	2.655530
O	-1.715651	1.822161	1.906593	C	-2.533511	2.688648	2.655485
C	2.530411	0.596189	0.550763	H	4.630593	1.090845	0.363499
C	3.705548	0.873137	-0.157996	H	4.615134	1.087789	-2.084956
C	3.694449	0.870835	-1.551154	H	2.526030	0.596018	-3.347681
C	2.529052	0.596441	-2.261607	H	0.000564	0.000980	-3.231293
C	1.369927	0.323248	-1.548613	H	-1.777148	1.890896	-3.348005
C	0.000414	0.000747	-2.138594	H	-3.248465	3.453580	-2.085487
C	-0.963776	1.025522	-1.548773	H	-3.260382	3.464458	0.362864
C	-1.779616	1.892914	-2.261929	H	-0.000130	-0.000237	1.533747
C	-2.600356	2.764673	-1.551579	H	-1.370627	-4.555453	0.360780
C	-2.608717	2.772608	-0.158424	H	-1.365720	-4.539014	-2.087751
C	-1.781556	1.893279	0.550498	H	-0.747036	-2.483131	-3.349135
C	-0.958892	1.019600	-0.155986	H	3.979956	1.854661	2.454628
C	0.000017	0.000055	0.447291	H	4.388452	0.112706	2.454058
C	1.362372	0.321180	-0.155829	H	-3.598602	2.514496	2.453591
C	-0.403117	-1.339965	-0.156552	H	-2.296795	3.741934	2.455319
C	-0.748984	-2.489479	0.549294	H	3.301392	0.776243	3.703469
C	-1.096750	-3.645160	-0.160216	H	-2.326280	2.467382	3.703357
C	-1.093270	-3.633544	-1.553350	O	-0.721267	-2.397609	1.905450
C	-0.748132	-2.486668	-2.263059	C	-1.064767	-3.539349	2.653096
C	-0.405115	-1.346724	-1.549321	H	-0.382206	-4.375323	2.451177

Table S3. Cartesian coordinates (in Å) of the optimized structure of 1,8-dimethoxytriptycene [RωB97X-D/6-31G(d)][S3] (Fig. 8b). The Gaussian16 software was used for the DFT calculations.[S4]

O	-1.956401	-1.262263	2.175456	C	2.153033	0.152711	0
O	-1.956401	-1.262263	-2.175456	C	-2.864471	-1.558921	3.209766
C	-1.251848	-0.101819	2.256758	C	-2.864471	-1.558921	-3.209766
C	-1.360951	0.821213	3.303401	H	-2.041145	0.647340	4.129274
C	-0.587890	1.980225	3.291329	H	-0.684867	2.685586	4.111489
C	0.300058	2.243308	2.251923	H	0.901415	3.147649	2.248998
C	0.399628	1.321830	1.218679	H	1.916074	2.334624	0
C	1.311437	1.424393	0	H	0.901415	3.147649	-2.248998
C	0.399628	1.321830	-1.218679	H	-0.684867	2.685586	-4.111489
C	0.300058	2.243308	-2.251923	H	-2.041145	0.647340	-4.129274
C	-0.587890	1.980225	-3.291329	H	-0.732683	-1.629762	0
C	-1.360951	0.821213	-3.303401	H	3.862946	-3.309544	0
C	-1.251848	-0.101819	-2.256758	H	5.233115	-1.249375	0
C	-0.367451	0.159145	-1.213210	H	4.135495	0.982050	0
C	-0.121282	-0.729647	0	H	-3.651578	-0.797527	3.286627
C	-0.367451	0.159145	1.213210	H	-2.356811	-1.651674	4.178735
C	1.376731	-1.012926	0	H	-2.356811	-1.651674	-4.178735
C	1.983578	-2.256998	0	H	-3.651578	-0.797527	-3.286627
C	3.379225	-2.337096	0	H	1.379106	-3.160054	0
C	4.149278	-1.179805	0	H	-3.316272	-2.516773	2.948371
C	3.534998	0.075918	0	H	-3.316272	-2.516773	-2.948371

4. Supporting References

S1 N. Seiki, Y. Shoji, T. Kajitani, F. Ishiwari, A. Kosaka, T. Hikima, M. Takata, T. Someya, T. Fukushima, *Science* 2015, **348**, 1122–1126.

S2 M. Kondo, T. Kajitani, T. Uemura, Y. Noda, F. Ishiwari, Y. Shoji, T. Araki, S. Yoshimoto, T. Fukushima, T. Sekitani, *Sci. Rep.* 2019, **9**, 9200.

S3 J.-D. Chai, M. Head-Gordon, *Phys. Chem. Chem. Phys.* 2008, **10**, 6615–6620.

S4 M. J. Frisch, G. W. Trucks, H. B. Schlegel, G. E. Scuseria, M. A. Robb, J. R. Cheeseman, G. Scalmani, V. Barone, G. A. Petersson, H. Nakatsuji, X. Li, M. Caricato, A. V. Marenich, J. Bloino, B. G. Janesko, R. Gomperts, B. Mennucci, H. P. Hratchian, J. V. Ortiz, A. F. Izmaylov, J. L. Sonnenberg, D. Williams-Young, F. Ding, F. Lipparini, F. Egidi, J. Goings, B. Peng, A. Petrone, T. Henderson, D. Ranasinghe, V. G. Zakrzewski, J. Gao, N. Rega, G. Zheng, W. Liang, M. Hada, M. Ehara, K. Toyota, R. Fukuda, J. Hasegawa, M. Ishida, T. Nakajima, Y. Honda, O. Kitao, H. Nakai, T. Vreven, K. Throssell, J. A., Jr. Montgomery, J. E. Peralta, F. Ogliaro, M. J. Bearpark, J. J. Heyd, E. N. Brothers, K. N. Kudin, V. N. Staroverov, T. A. Keith, R. Kobayashi, J. Normand, K. Raghavachari, A. P. Rendell, J. C. Burant, S. S. Iyengar, J. Tomasi, M. Cossi, J. M. Millam, M. Klene, C. Adamo, R. Cammi, J. W. Ochterski, R. L. Martin, K. Morokuma, O. Farkas, J. B. Foresman, D. J. Fox, Gaussian, Inc., Gaussian 16, Revision B.01, Wallingford CT, 2016.
